# Human bone marrow-derived mesenchymal stromal cells cultured in serum-free media demonstrate enhanced antifibrotic abilities via prolonged survival and robust regulatory T cell induction in murine bleomycin-induced pulmonary fibrosis

**DOI:** 10.1186/s13287-021-02574-5

**Published:** 2021-09-16

**Authors:** Shun Takao, Taku Nakashima, Takeshi Masuda, Masashi Namba, Shinjiro Sakamoto, Kakuhiro Yamaguchi, Yasushi Horimasu, Shintaro Miyamoto, Hiroshi Iwamoto, Kazunori Fujitaka, Hironobu Hamada, Shinya Takahashi, Ayumu Nakashima, Noboru Hattori

**Affiliations:** 1 Department of Molecular and Internal Medicine, Graduate School of Biomedical and Health Sciences, Hiroshima University, 1-2-3, Kasumi, Minami-ku, Hiroshima, 734-8551 Japan; 2 Department of Clinical Oncology, Hiroshima University Hospital, 1-2-3, Kasumi, Minami-ku, Hiroshima, 734-8551 Japan; 3 Department of Physical Analysis and Therapeutic Sciences, Graduate School of Biomedical and Health Sciences, Hiroshima University, 1-2-3 Kasumi, Minami-ku, Hiroshima, 734-8551 Japan; 4 Department of Cardiovascular Surgery, Graduate School of Medicine, Hiroshima University, 1-2-3, Kasumi, Minami-ku, Hiroshima, 734-8551 Japan; 5 Department of Stem Cell Biology and Medicine, Graduate School of Biomedical and Health Sciences, Hiroshima University, 1-2-3 Kasumi, Minami-ku, Hiroshima, 734-8553 Japan

**Keywords:** Mesenchymal stromal cells, Serum-free, Bleomycin, Pulmonary fibrosis, Regulatory T cells, Interleukin-6, Transforming growth factor-β

## Abstract

**Background:**

Mesenchymal stromal cells (MSCs) are a potential therapeutic tool for pulmonary fibrosis. However, ex vivo MSC expansion using serum poses risks of harmful immune responses or unknown pathogen infections in the recipients. Therefore, MSCs cultured in serum-free media (SF-MSCs) are ideal for clinical settings; however, their efficacy in pulmonary fibrosis is unknown. Here, we investigated the effects of SF-MSCs on bleomycin-induced pulmonary inflammation and fibrosis compared to those of MSCs cultured in serum-containing media (S-MSCs).

**Methods:**

SF-MSCs and S-MSCs were characterized in vitro using RNA sequence analysis. The in vivo kinetics and efficacy of SF-MSC therapy were investigated using a murine model of bleomycin-induced pulmonary fibrosis. For normally distributed data, Student’s t test and one-way repeated measures analysis of variance followed by post hoc Tukey’s test were used for comparison between two groups and multiple groups, respectively. For non-normally distributed data, Kruskal–Wallis and Mann–Whitney U tests were used for comparison between groups, using e Bonferroni’s correction for multiple comparisons. All tests were two-sided, and *P* < 0.05 was considered statistically significant.

**Results:**

Serum-free media promoted human bone marrow-derived MSC expansion and improved lung engraftment of intravenously administered MSCs in recipient mice. SF-MSCs inhibited the reduction in serum transforming growth factor-β1 and the increase of interleukin-6 in both the serum and the bronchoalveolar lavage fluid during bleomycin-induced pulmonary fibrosis. SF-MSC administration increased the numbers of regulatory T cells (Tregs) in the blood and lungs more strongly than in S-MSC administration. Furthermore, SF-MSCs demonstrated enhanced antifibrotic effects on bleomycin-induced pulmonary fibrosis, which were diminished by antibody-mediated Treg depletion.

**Conclusions:**

SF-MSCs significantly suppressed BLM-induced pulmonary inflammation and fibrosis through enhanced induction of Tregs into the lungs and corrected the dysregulated cytokine balance. Therefore, SF-MSCs could be a useful tool for preventing pulmonary fibrosis progression without the demerits of serum use.

**Supplementary Information:**

The online version contains supplementary material available at 10.1186/s13287-021-02574-5.

## Background

Idiopathic pulmonary fibrosis (IPF) is a severe pulmonary fibrotic disease that presents with short life expectancy and a high mortality rate [1]. Since patients with IPF who are treated with antifibrotic agents show inhibition of forced vital capacity (FVC) decline and improved survival, antifibrotic agents such as pirfenidone and nintedanib have been conditionally recommended for IPF treatment [2]. However, these agents cannot halt disease progression; moreover, they have adverse effects, including gastrointestinal disorders, skin-related problems, and liver damage [3]. Thus, considering the lack of more effective options for treating IPF, further therapeutic approaches are being explored.

Mesenchymal stromal cells (MSCs) are pluripotent cells in the bone marrow and are now known to be isolated from various sources, including adipose tissue, umbilical cord, peripheral blood, and muscle tissue [4]. Systemically administered MSCs home to the site of injury and exert anti-inflammatory effects by modulating various immune cells. Furthermore, MSCs secrete cytokines and growth factors with proliferative and angiogenic effects and support tissue repair through paracrine effects [5]. With the expectation of applying these favorable effects to therapy, several preclinical and clinical studies are ongoing using human MSCs to treat chronic diseases including autoimmune, inflammatory, degenerative, and cardiovascular diseases [6]. Regarding lung diseases, MSC-based therapies have been reported as effective in preventing experimental models of pulmonary fibrosis [7]. Based on successful studies with animal models, clinical trials of MSC-based therapies for interstitial lung diseases, mainly human IPF, are underway worldwide [8]. Some clinical trials have shown that MSC-based cell therapy provides a protective effect against FVC decline over time in patients with IPF [9, 10]. In addition, no serious adverse events related to MSC-based cell therapy have been reported in these trials, suggesting the safety and tolerance of this therapy [8, 10]. These findings suggest that MSC-based cell therapy could be a new potential therapeutic option for treating IPF.

In vitro expansion of MSCs is necessary before their transplantation into recipients of MSC-based therapies. MSC growth in vitro generally requires culture media supplemented with fetal bovine serum (FBS) or human serum to provide the factors essential for cell growth. In fact, most studies on MSCs have used serum-containing media [11]. However, using serum in culture media poses various potential disadvantages including pathogen contamination (e.g., unknown viruses, mycoplasma, and prions), harmful immunizing effects [11, 12], inhibition of cell growth [13], uneven quality between lots, global shortage of supply, and high costs [14]. Therefore, defined culture conditions without sera are ideal as a tool for MSC therapy in humans. Recently, several chemically defined serum-free media for experimental MSC cultures have been commercialized [11, 14]. Wu et al. showed that human MSCs cultured in serum-free medium (SF-MSCs) exhibited strong immunomodulatory activity and secreted higher levels of immunoregulatory factors compared with MSCs cultured in serum-containing medium (S-MSCs) in vitro, and showing improved therapeutic activity in a rat model of pulmonary arterial hypertension in vivo [15]. Moreover, other animal studies have also reported better therapeutic efficacy of SF-MSCs compared with that of S-MSCs in a mouse model of acute colitis [16], a rat model of renal fibrosis [17] and peritoneal fibrosis [18]. Thus, using SF-MSCs not only avoids the disadvantages of using sera, but SF-MSCs may also be more useful as a therapeutic tool than S-MSCs. Previous preclinical studies of SF-MSCs used for lung disease reported that xenogeneic SF-MSCs demonstrated therapeutic effects in a rat model of *Escherichia coli*-induced [19] and ventilator-induced lung injury [20]. However, the therapeutic effects of SF-MSCs on pulmonary fibrosis have not been investigated. Considering the progress of IPF clinical trials with MSCs, clarifying the efficacy of SF-MSCs in experimental pulmonary fibrosis is essential for developing MSC-based therapies in human IPF. Therefore, in the current study, we investigated the effects of SF-MSCs on an experimental mouse model of lung fibrosis and compared these effects with those of S-MSCs.

## Methods

### MSCs

Human bone marrow-derived MSCs were collected from the sternum of consenting patients during thoracic surgery with the approval of the Ethics Committee of Hiroshima University Hospital (E-1089). MSCs were cultured in Dulbecco’s modified Eagle’s medium (DMEM) (Sigma-Aldrich, St. Louis, MO, USA) supplemented with 10% FBS (Sigma-Aldrich) for S-MSCs, or with serum-free STK2 medium (KBDSTC102; DS Pharma Biomedical) for SF-MSCs. The MSCs were dissociated with Accutase (Innovative Cell Tech, San Diego, CA, USA) and passaged 5–6 times before use. For the proliferation assay, MSCs were seeded into 24-well plates at a density of 5 × 10^3^ cells/well and cultured as S-MSCs or SF-MSCs. After staining with Trypan blue (Sigma, St. Louis, MO, USA), the cell number per well was counted at 24, 48, 72, 96, and 120 h after the start of the cell culture using an automated cell counter (TC-20, Bio-Rad, Hercules, CA, USA). Cell morphologies were also recorded at the indicated time points using a Nikon Diaphot 300 microscope (Nikon, Tokyo, Japan). For the measurement of cytokine secretion by MSCs, MSCs pre-cultured in DMEM with 10% FBS (S-MSCs) or with serum-free STK2 medium (SF-MSCs) were seeded into 24-well plates at a density of 5 × 10^3^ cells/well. They were cultured in serum-free DMEM for 48 h. During MSCs incubation, the culture supernatants were collected and cleared of cells by centrifugation for 10 min at 300 × *g* at 10 °C. The supernatants were stored at − 80 °C and used for measuring the cytokine concentrations.

### Flow cytometry analysis

To obtain single-cell suspensions, mice were euthanized, and the middle and lower lobes of the right lung, spleen, or thymus were excised, minced, and digested in RPMI 1640 medium containing 1.0 mg/mL collagenase A (Roche Diagnostics, Basel, Switzerland) and 20 U/mL DNase I (Takara Bio Inc., Shiga, Japan) at 37 °C for 30 min. Red blood cells (RBCs) were lysed using ACK lysis buffer (Thermo Fisher Scientific, Waltham, MA, USA). Murine blood samples for flow cytometry analysis were subjected to RBC lysis twice, according to the ACK lysis buffer protocol. After blocking with anti-mouse CD16/32 Abs (FcγR, clone 93, BioLegend, San Diego, CA, USA), cell suspensions were incubated with appropriate dilutions of antibodies or their isotype-matched controls. Rat monoclonal antibodies for mouse CD3 (17A2), CD4 (RM4-5), CD25 (PC61), and mouse monoclonal antibodies against human CD11b (ICRF44), CD19 (SJ25C1), CD34 (581), CD44 (IM7), CD45 (HI30), CD73 (AD2), CD90 (5E10), CD105 (SN6h), and HLA-DR (L243) were purchased from BioLegend. For intracellular staining, cells were fixed and permeabilized with a Cytofix/Cytoperm Kit (BD Biosciences, San Jose, CA, USA) before staining with FoxP3 (clone MF-14, BioLegend). CD3^+^/CD4^+^/CD25^+^/FoxP3^+^ cells were defined as regulatory T cells. Cells were analyzed on the BD FACS Aria II (BD Biosciences) or the BD LSR Fortessa X-20 system (BD Biosciences). The collected data were analyzed using the FlowJo software (Tree Star, Inc., Ashland, OR, USA).

### RNA extraction and sequencing

Total RNA from S-MSCs and SF-MSCs was extracted using the RNeasy Mini Kit (Qiagen, Hilden, Germany), according to the manufacturer’s protocol. Extracted RNA was quantified and qualified using an Agilent 2100 Bioanalyzer (Agilent Technologies, Santa Clara, CA, USA) according to the manufacturer’s instructions. Total RNA (1 µg) with an RNA Integrity Number value > 8 was used for library construction, which was done using a SMART-Seq Stranded Kit (Takara Bio, Shiga, Japan). The qualified libraries were sequenced using an Illumina Hiseq 2500 system (Illumina, CA, USA) with single-end reads. The raw reads were aligned against the whole genome build hg19 using the StrandNGS v2.7 software (Strand Genomics, Inc., San Francisco, CA, USA).

### Enrichment analysis of differentially expressed genes

Gene Ontology (GO) enrichment analysis and Kyoto Encyclopedia of Genes and Genomes (KEGG) pathway analysis were performed to explore the biological functions of the differentially expressed genes (DEGs) between S-MSCs and SF-MSCs. Transcripts with fold-change values greater than 2.0, with a *P* ≤ 0.05, were included in the analysis as DEGs. In GO enrichment analysis, these DEGs were assigned to one of three categories: biological processes (BP), molecular functions (MF), and cellular components (CC). Enrichment analyses of the DEGs were carried out using the Database for Annotation, Visualization, and Integrated Discovery v.6.8 online software tool (<http://david.abcc.ncifcrf.gov/>).

### DiI labeling

MSCs were labeled using CellTracker CM-DiI (Thermo Fisher Scientific) according to the manufacturer’s protocols before intravenous injection. Briefly, the DiI solution stock was diluted with dimethyl sulfoxide (DMSO, Sigma-Aldrich) at a concentration of 2 mg/mL to prepare the DiI working solution. Labeling was performed by adding the DiI working solution to the cell suspension at a final concentration of 5 µg/mL and incubating for 25 min at 37 °C with 5% CO_2_. After labeling, the cells were washed with fresh DMEM.

### Animals

Male C57BL/6 mice (6–8 weeks old) were purchased from Charles River Laboratories Japan (Yokohama, Japan), housed in pathogen-free rooms with a controlled environment under a 12-h light–dark cycle, and maintained on laboratory chow with free access to food and water. All experimental procedures were approved by the Committee on Animal Research at Hiroshima University (Approval No. A17-28) and were conducted under the *Guide for the Care and Use of Laboratory Animals*, *8th ed*, 2010 (National Institutes of Health, Bethesda, MD, USA).

### Oropharyngeal aspiration (OA) of bleomycin (BLM) in mice

Mice were first anesthetized using mixed anesthetic agents, including medetomidine (0.3 mg/kg body weight; Kyoritsu Seiyaku, Tokyo, Japan), midazolam (4 mg/kg body weight, Sandoz K.K., Tokyo, Japan), and butorphanol (5 mg/kg body weight, Meiji Seika Pharma, Tokyo, Japan). They were then administered BLM (Nippon Kayaku, Tokyo, Japan) at a dose of 2.0 mg/kg body weight in phosphate-buffered saline (PBS) via OA using a micropipette. The OA procedure was performed as described previously [21]. Briefly, mice were secured on a platform, their tongue was pulled out with forceps, and the BLM solution was placed onto the distal part of the oropharynx, while the nasal cavity was closed gently with the technician’s fingers.

### MSC administration

SF-MSCs or S-MSCs (1 × 10^5^ cells/mouse) in 100 µL of PBS were injected through the tail vein at 4 days after BLM OA. In the PBS group, 100 µL of PBS alone was injected through the tail vein. For analyzing cell engraftment, DiI-labeled MSCs were injected into the mice via the tail vein at a dose of 2.0 × 10^5^ cells/mouse in 100 µL of PBS. Control mice were injected with unlabeled SF-MSCs at the same dose through the tail vein.

### Immunohistochemical staining

Immunohistochemical staining was performed on paraffin-embedded tissues to evaluate the retention of MSCs. Briefly, the tissue sections were incubated overnight at 4 °C with the anti-human nuclei mouse monoclonal antibody (3E1.3, MAB 4383, MilliporeSigma, Burlington, MA, USA) diluted in 0.05 M phosphate buffer (pH 7.6). A Histo-fine simple stain MAX-PO (Multi) kit (Nichirei, Tokyo, Japan) was used to detect antigen binding. Nuclear staining was performed using Mayer’s hematoxylin.

### Hydroxyproline assay

For the biochemical analysis of lung fibrosis, murine left lungs were evaluated for hydroxyproline content. Briefly, at 7 or 14 days after BLM OA, the left lung was removed and the extrapulmonary airways and blood vessels were excised and discarded. After homogenization in 1.0 mL of PBS, 1.0 mL of 12 N HCl was added, and the samples were hydrolyzed at 120 °C for 16 h. In a 96-well plate, 5 μL of each sample was combined with 5 μL of citrate/acetate buffer (5% citric acid, 1.2% glacial acetic acid, 7.25% sodium acetate, and 3.4% sodium hydroxide). Then, 100 µL of chloramine-T solution (0.141 g of chloramine-T added to 8 mL of citrate/acetate buffer, 1.0 mL of n-propanol, and 1.0 mL of Milli-Q water) was added, and the mixture was incubated for 30 min 25 °C. After this incubation, 100 µL of Ehrlich’s solution (1.25 g of p-dimethylaminobenzaldehyde added to 4.65 mL of n-propanol and 1.95 mL of 70% perchloric acid) was added and the samples were incubated at 65 °C for 30 min. The absorbance of each sample was then measured at 540 nm wavelength. Standard curves were generated for each experiment using hydroxyproline as the standard reagent. Results are expressed as micrograms of hydroxyproline in the left lung tissue.

### Histological analysis of lung fibrosis

The right lungs were inflation-fixed with a buffered 4% formalin solution. After embedding in paraffin, the sections were stained with hematoxylin and eosin (H&E).

### Analysis of bronchoalveolar lavage fluid (BALF)

BALF was collected before BLM OA, or at 7 or 14 days after BLM OA. Briefly, after euthanasia, the murine tracheas were exposed and cannulated using an 18-gauge cannula, and the lungs were lavaged thrice with 0.5 mL of PBS. Lavage fluids were pooled and cleared of cells by centrifugation for 5 min at 300 × *g* at 4 °C. The supernatants were stored at − 80 °C and used for measuring the cytokine concentrations. The cell pellets were resuspended in 1 mL of DMEM, and the total cell numbers were determined using an automated cell counter after red blood cell lysis using the ACK lysis buffer. Differential cell counts were obtained with the Diff-Quik stain (Kokusai Shiyaku, Kobe, Japan) using Cytospin (Thermo Fisher Scientific).

### Cytokine measurements

Cytokine levels including free active transforming growth factor (TGF)-β1, granulocyte macrophage-colony-stimulating factor (GM-CSF), interferon (IFN)-γ, tumor necrosis factor (TNF)-α, interleukin (IL)-2, IL-4, IL-5, IL-6, IL-10, IL-13, and IL-33, in mouse bronchoalveolar lavage fluid (BALF) and serum were assessed using the LEGENDplex (BioLegend) custom panel assay kit as per the manufacturer’s instructions. Cytokine levels were determined using a FACSverse flow cytometer (BD Biosciences), and analyses were performed using the LEGENDplex data analysis software (BioLegend). Total TGF-β1 protein in the culture supernatant or in MSCs was measured using a Human TGF-β1 Quantikine ELISA kit (R&D Systems, Minneapolis, MN, USA) following the manufacturer’s instructions.

### Blood sampling

Blood samples for flow cytometry analysis were collected at 6, 10, or 14 days after BLM OA from the facial veins of live anesthetized mice. Blood samples for cytokine measurement were collected at 7, 14, or 21 days after BLM OA by right ventricular cardiac puncture at the time of sacrifice. Blood samples for serum cytokine analysis were cleared of cells by centrifugation at 1000 × *g* for 15 min at 4 °C. The supernatants were stored at − 80 °C and used for measuring the cytokine concentrations.

### Lung homogenate co-culture and protein extraction from MSCs

Fresh left lungs harvested from mice treated with PBS OA or BLM OA at a dose of 2.0 mg/kg body weight were homogenized in 1.0 mL of DMEM or STK2 using a handheld homogenizer. Then, 100 μL of lung homogenate was added onto co-culture inserts for 6-well plates with a 0.4 μm pore size porous polyester membrane (Corning, NY, USA). The well inserts were combined in a 6-well culture plate pre-plated with 1.0 × 10^5^ MSCs. They were cultured in DMEM with 10% FBS (S-MSCs) or in serum-free STK2 medium (SF-MSCs). After co-culture for 72 h, MSCs were lysed with Nonidet P-40 (NP-40) lysis buffer (50 mM Tris–HCl [pH 8.0], 150 mM NaCl, 1% NP-40) containing a protease inhibitor cocktail for measurement of TGF-β1 protein in MSCs.

### TGF-β1 administration

Mice were injected intraperitoneally with 400 ng of recombinant TGF-β1 (BioLegend) three times, at 3, 6, and 9 days after BLM OA.

### Regulatory T cell (Treg) depletion

To deplete Tregs, mice were injected twice intraperitoneally with 30 μg of purified anti-mouse CD25 antibody (clone PC61, BioLegend) in 200 µL of PBS, at 6 and 9 days after BLM OA (i.e., at 2 and 5 days following SF-MSC injections). The control group was treated with rat isotype IgG1 (BioLegend).

### Statistical analyses

Statistical analyses were performed using the JMP Pro 15 software (SAS Institute Inc., Cary, NC, USA). The results are expressed as mean ± standard deviation (SD) for normal distribution and median with interquartile range for non-normal distribution. In normal distribution data analyses, Student’s *t* test (two-tailed paired or unpaired) was performed for comparison between two groups, and one-way repeated measures analysis of variance (ANOVA) followed by the *post hoc* Tukey’s test was performed for comparison between multiple groups. In the analysis of non-normally distributed data, the Kruskal–Wallis and Mann–Whitney U tests were used for comparison between groups, using Bonferroni’s correction for multiple comparisons. All tests were two-sided, and *P* < 0.05 was considered statistically significant.

## Results

### Comparison of characteristics between SF-MSCs and S-MSCs

**Fig. 1:**
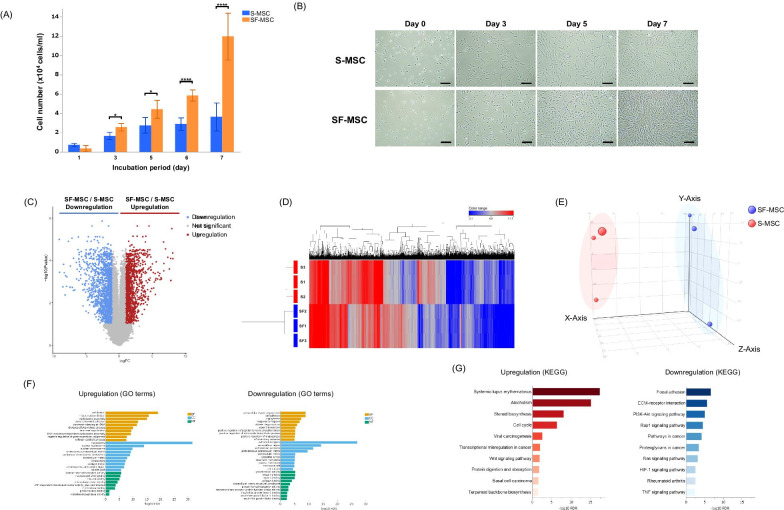
Effect of culture medium differences on MSCs proliferation. **A** The number of MSCs cultured in Dulbecco’s modified Eagle’s medium (DMEM) with 10% fetal bovine serum (S-MSC) or serum-free medium STK2 (SF-MSC). Data are presented as means ± SD (*n* = 4 per group). *p < 0.05. ****P < 0.001. **B** Microscopic images showing proliferation of S-MSCs and SF-MSCs. Scale bar, 200 µm. **C** Volcano plot showing the gene expression profiles of S-MSCs and SF-MSCs (*n* = 3 per group). Upregulated (FC ≥ 2.0, FDR *P* < 0.05) and downregulated (FC ≤ − 2.0, FDR *p* < 0.05) differentially expressed genes (DEGs) are marked by red and blue dots, respectively. The gray dots represent insignificant DEGs. **D** Hierarchical clustered heatmap showing the gene expression patterns of DEGs (FC ≤ -2.0, or ≥ 2.0; *P* < 0.05). Each row and line represent one sample and one DEG, respectively. Red and blue colors indicate upregulation and downregulation, respectively. **E** In the principal component analysis (PCA), each dot represents one MSC sample. The distance between the dots indicates the level of difference between the gene expression profiles of the samples. Dots with the same color created two distinct groups (red: S-MSC and blue: SF-MSC), and the distance between the differentially colored groups was prominent. **F** Upregulated and downregulated terms in Gene Ontology (GO) enrichment pathway analysis when comparing SF-MSCs with S-MSCs. The terms are categorized into biological process (BP), cellular component (CC), and molecular function (MF). The y-axis shows the top 10 terms, and the x-axis shows the negative logarithm of the *p* value. **G** The significantly enriched KEGG pathways (*P* < 0.05) are presented. For each KEGG pathway, the bar shows fold enrichment of the pathway. The y-axis indicates the top 10 pathway categories, and the x-axis indicates the -log10 of the false discovery rate (FDR)-adjusted *p* value

We initially compared the proliferative ability of SF-MSCs with that of S-MSCs. Compared with that in serum-containing medium, the number of MSCs was significantly increased in serum-free medium (Fig. 1A, B). To determine whether SF-MSCs retained their expression of MSC surface biomarkers, we analyzed the expression of cell surface proteins, proposed as the minimal criteria for human MSCs by the International Society for Cellular Therapy (ISCT) [22], in SF-MSCs and S-MSCs using flow cytometry. Both S-MSCs and SF-MSCs were negative for CD11b, CD19, CD34, CD45, and HLA-DR and were positive for CD44, CD73, CD90, and CD105 (Additional file 1). In RNA sequencing analysis, out of 16,806 quantitatively detected genes, 2,417 DEGs were identified between the two MSC populations, including 1,120 significantly upregulated DEGs and 1,297 significantly downregulated DEGs, when comparing SF-MSCs with S-MSCs (Fig. 1C). The clustering analysis in both directions between the DEGs provided evidence on whether the two types of MSCs were different (Fig. 1D). Similarly, principal component analysis (PCA) showed that the groups were far from each other, indicating a difference in gene expression between the S-MSCs and SF-MSCs (Fig. 1E). To analyze the functions of these DEGs, data from the GO database and the KEGG database were used to perform pathway analysis. In GO term analysis, the upregulated DEGs were mainly associated with cell proliferation and the downregulated DEGs were mainly associated with extracellular components (Fig. 1F). In the BP category, positive regulation of apoptotic process (GO:0043065) was downregulated in SF-MSCs compared with S-MSCs (− log10 FDR = 0.004. Not shown in Fig. 1F due to being ranked outside of top 10 downregulated terms). In KEGG pathway analysis, several downregulated pathways were related to bleomycin-induced pulmonary fibrosis including PI3K/AKT signaling, renin angiotensin system (RAS) signaling, and hypoxia-inducible factor 1 (HIF-1) signaling (Fig. 1G) [23–25]. Further, the cell cycle pathway was upregulated, indicating the high proliferative potential of SF-MSCs.

### Increased murine lung engraftment of MSCs cultured in serum-free medium

**Fig. 2:**
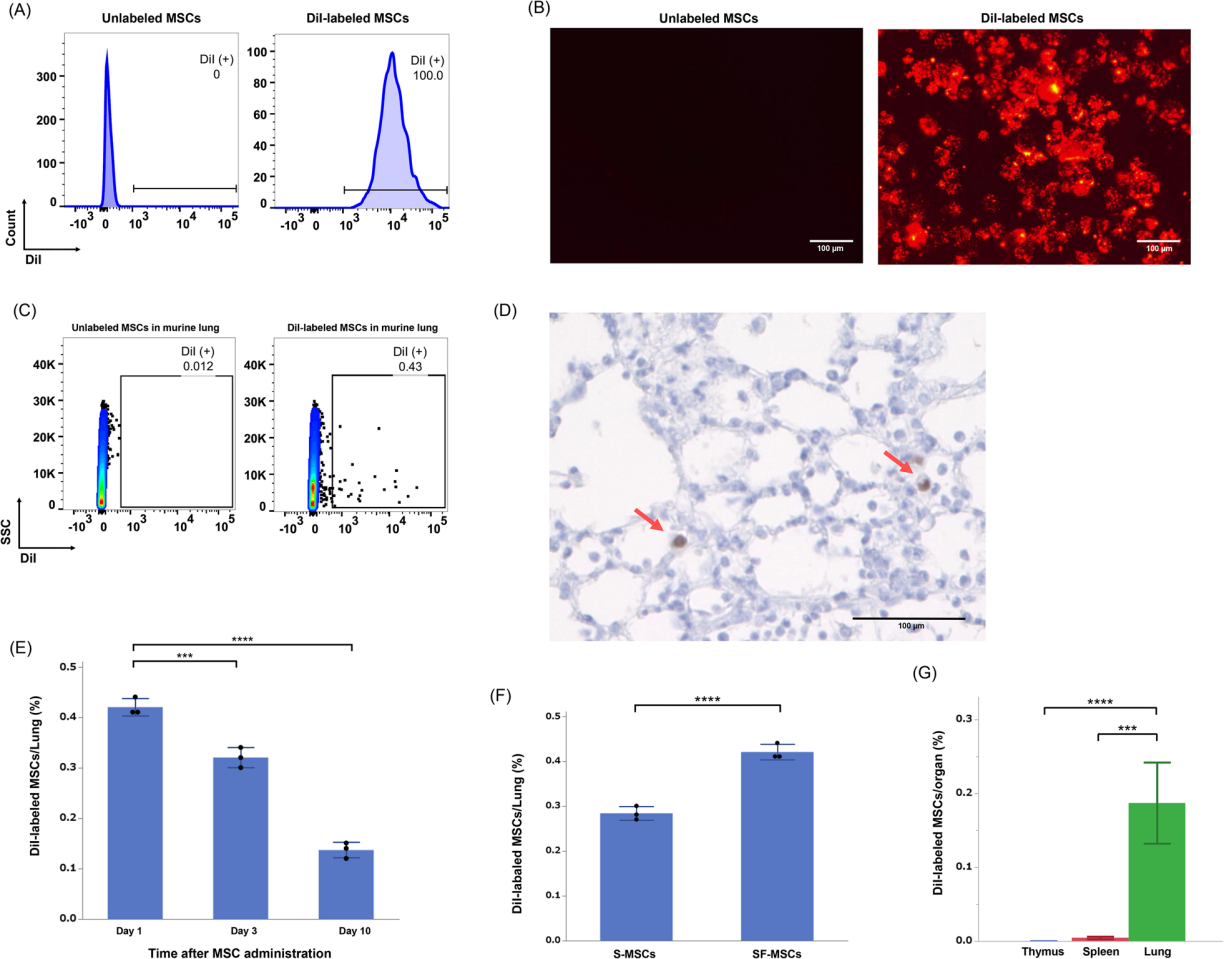
Engraftment of administered MSCs in murine lungs. **A** MSCs were labeled with CellTracker CM-DiI before injection. DiI-labeled MSCs were detected as the positive fraction in the phycoerythrin (PE) channel using flow cytometry. **B** Fluorescence microscopy images showing DiI-labeled MSCs with red fluorescence. **C** MSCs engrafted in murine lungs were detected using flow cytometry on the day after intravenous SF-MSC administration (i.e., at 5 days after BLM OA). **D** Confirmation of engrafted human-derived MSCs in murine lung tissues using anti-human nuclear antibody staining (red arrows). **E** At 1, 3, or 10 days after DiI-labeled SF-MSC injection (i.e., at 5, 7, or 14 days after BLM OA), the percentages of engrafted SF-MSCs in the lungs were measured (*n* = 3 per group). ***P < 0.005. ****P < 0.001. **F** The percentages of SF-MSCs or S-MSCs engrafted in murine lungs on the day after DiI-labeled MSC injection (*n* = 3 per group). ****P < 0.001. **G** The percentages of SF-MSCs engrafted in the murine thymus, spleen, or lung on the day after DiI-labeled SF-MSC injection. The mice in the PBS group were administered only 100 µL of PBS (*n* = 3 per group). Data are presented as means ± SD

To evaluate the number of cells engrafted in murine lungs, MSCs were labeled with CellTracker CM-DiI before intravenous administration to mice (Fig. 2A, B). DiI-labeled SF-MSCs were detected in murine lungs using flow cytometry (Fig. 2C) and fluorescence microscopy (Additional file 2). Staining with an anti-human nuclear antibody confirmed the engraftment of SF-MSCs in the murine lungs (Fig. 2D). Flow cytometry analysis revealed a decrease in the number of SF-MSCs engrafted into the murine lungs over time (Fig. 2E). The number of MSCs engrafted in the murine lungs on the day after MSCs injection was 50% higher in SF-MSCs (0.42% ± 0.02%) than S-MSCs (0.28% ± 0.02%) transplanted recipients (Fig. 2F). We also evaluated the number of SF-MSCs engrafted into the murine thymus or spleen after intravenous MSC administration and found that few MSCs were engrafted into either organ (Fig. 2G).

### Enhanced antifibrotic and anti-inflammatory effect of SF-MSCs in BLM-induced pulmonary fibrosis

**Fig. 3:**
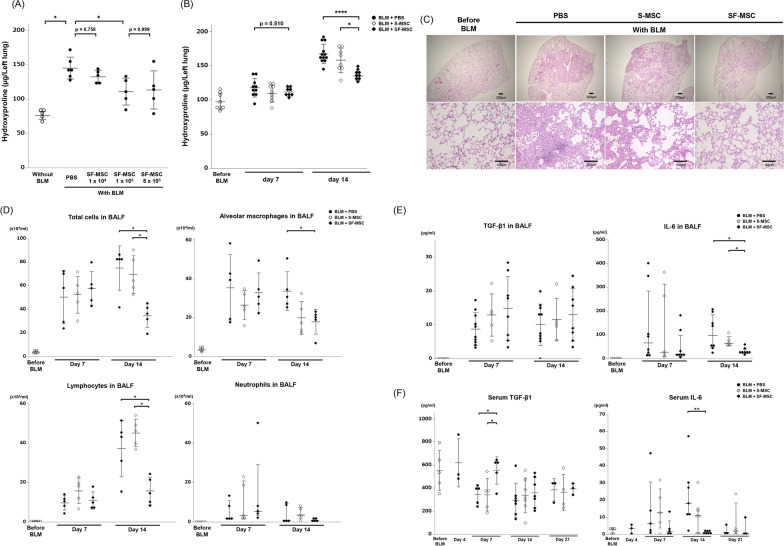
Antifibrotic effect of SF-MSCs in BLM-induced pulmonary fibrosis in mice. **A** At 4 days after BLM OA, SF-MSCs were injected via the tail vein at a dose of 1 × 10^4^, 1 × 10^5^, or 5 × 10^5^ cells in 100 µL of PBS. Mice in the without-BLM group were aspirated with 100 µL of PBS alone instead of BLM and were injected with 100 µL of PBS intravenously. At 14 days after BLM OA, the hydroxyproline levels in the murine left lung were measured (*n* = 5 per group). **B** The hydroxyproline levels in the murine left lung before or at 7 or 14 days after BLM OA (i.e., at 3 or 10 days after intravenous MSC administration) (*n* = 8–12 per group). **C** Histological analyses using hematoxylin and eosin staining in lung sections obtained before or at 14 days after BLM OA. **D** Inflammatory cells in BALF were measured before or at 7 or 14 days after BLM OA (*n* = 5 per group). **E** Cytokine levels in BALF before or at 7 or 14 days after BLM OA (*n* = 5–10 per group). **F** Cytokine levels in the serum before or at 4, 7, 14, or 21 days after BLM OA (*n* = 3–7 per group). Data are presented as the mea*n* ± SD for normal distribution, or as the median with interquartile range for non-normal distribution. *P < 0.05. **P < 0.01. ****P < 0.001

To determine the optimal dose of intravenous SF-MSC injection, we compared different doses of SF-MSCs and assessed their antifibrotic effects on BLM-induced pulmonary fibrosis. No significant difference in lung hydroxyproline levels was found between SF-MSCs in the 1 × 10^4^ and PBS groups. SF-MSCs administered at a dose of over 1 × 10^5^ cells significantly inhibited the elevation of lung hydroxyproline levels, whereas there was no difference between the 1 × 10^5^ and 5 × 10^5^ groups (Fig. 3A). Therefore, we used a dose of 1 × 10^5^ cells as the therapeutic dose. Next, we compared the therapeutic effects of SF-MSCs and S-MSCs. The hydroxyproline levels in the SF-MSC group were significantly lower than those in the S-MSC group on day 14 (Fig. 3B). H&E staining of lung tissues also confirmed reduced patches of lung fibrotic areas, as evidenced by thickening of the alveolar septa and inflation of the alveoli in the SF-MSC group when compared with the S-MSC group (Fig. 3C). Like the above experiments, SF-MSCs, but not S-MSCs, significantly suppressed the number of total cells, macrophages, and lymphocytes in the BALF at 14 days after BLM OA (Fig. 3D). In BALF cytokine analysis, SF-MSC treatment suppressed the BLM-induced increase in IL-6, but not in TGF-β1 (Fig. 3E). The BALF levels of TNF-α, IL-4, IL-5, IL-10, and IL-13 were not significantly different between the groups (Additional file 3). In serum cytokine analysis, the level of TGF-β1 gradually decreased with a nadir at 14 days after BLM OA; this decrease was suppressed in the SF-MSC group at 7 days after BLM OA. Conversely, the serum level of IL-6 gradually increased with a peak at 14 days after BLM OA; this increase was abolished in the SF-MSC group at 7 days after BLM OA (Fig. 3F). Meanwhile, the serum levels of IFN-γ, TNF-α, GM-CSF, IL-2, IL-4, IL-5, IL-10, IL-13, and IL-33 were not significantly different between the groups (Additional file 4). BLM administration caused murine weight loss, which was not inhibited by treatment of SF-MSCs (Additional file 5). Interestingly, subcutaneous and intraperitoneal administration of SF-MSCs did not demonstrate the antifibrotic effects and lung engraftment that were observed with the intravenous administration of SF-MSCs (Additional file 6 and 7).

### Increased numbers of Tregs in the blood and lungs after SF-MSCs treatment

**Fig. 4:**
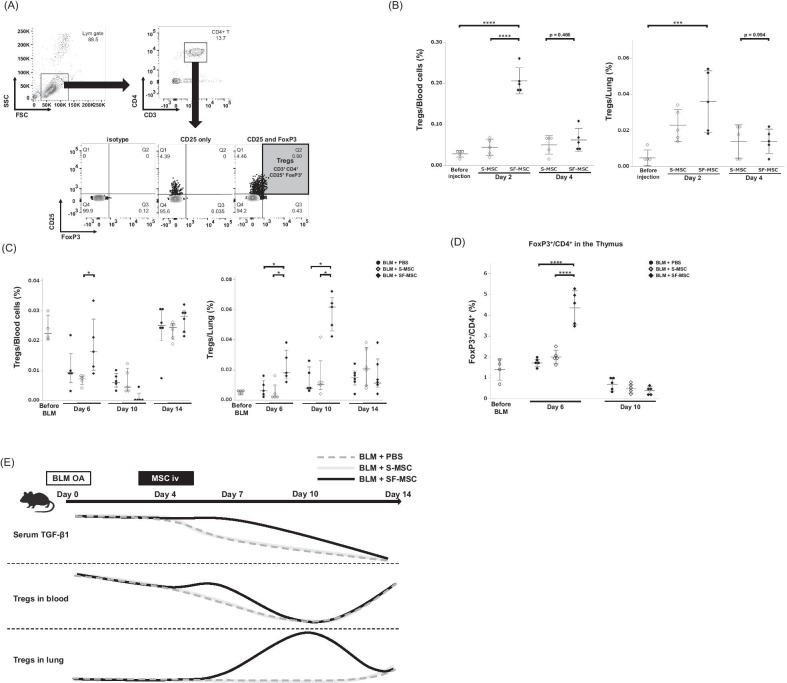
SF-MSC treatment enhanced Treg induction. **A** Representative flow cytometry analysis for the detection of Tregs in murine peripheral blood. Gated on lymphocytes in peripheral blood nucleated cells (upper left panel). Gated on CD3^+^/CD4^+^ T cells in the lymphocytes (upper right panel). Gray-filled gate indicates　CD3^+^/CD4^+^/CD25^+^/Foxp3^+^ Tregs (lower panel). **B** The percentages of Tregs in murine blood or lungs before or at 2 or 4 days after MSC injection to healthy mice (*n* = 5 per group). **C** The percentages of Tregs in murine blood or lung before or at 6, 10, or 14 days after BLM OA followed by injection of MSCs (i.e., at 2, 6, or 10 days after MSCs injection) or PBS (i.e., at 2, 6, or 10 days after PBS injection) (*n* = 4–6 per group). **D** The percentages of FoxP3^+^/CD4^+^ T cells in the murine thymus before or at 6 or 10 days after BLM OA (*n* = 4–5 per group). Data are presented as the mean ± SD for normal distribution, or the median with interquartile range for non-normal distribution. *P < 0.05. ***P < 0.005. ****P < 0.001. **E** Schematic summary of the dynamics of serum TGF-β1, number of Tregs in the blood and lungs in BLM-treated mice followed by SF-MSC or PBS intravenous administration

Since the SF-MSC treatment inhibited the increased IL-6 and decreased TGF-both of which are associated with Treg induction, we hypothesized that SF-MSC treatment could induce differentiation of Tregs in BLM-treated mice and prevent lung fibrosis. We defined CD3^+^/CD4^+^/CD25^+^/FoxP3^+^ cells as Tregs (Fig. 4A) and found a significant increase in the number of blood and lung Tregs in healthy mice treated with SF-MSCs (Fig. 4B). Notably, SF-MSCs, but not S-MSCs, increased the number of Tregs during BLM-induced pulmonary fibrosis (Fig. 4C). In addition, the number of Foxp3^+^/CD4^+^ T cells in the thymus (virtually all thymic cells were positive for CD3) was transiently increased with SF-MSC treatment, suggesting that Treg differentiation was promoted in the thymus (Fig. 4D). The changes over time in serum TGF-β1, blood Tregs, and lung Tregs during BLM-induced pulmonary fibrosis treated with or without SF-MSC treatment are summarized in Fig. 4E.

### Effect of upregulated circulating TGF-β1 on Treg induction and BLM-induced pulmonary fibrosis

**Fig. 5:**
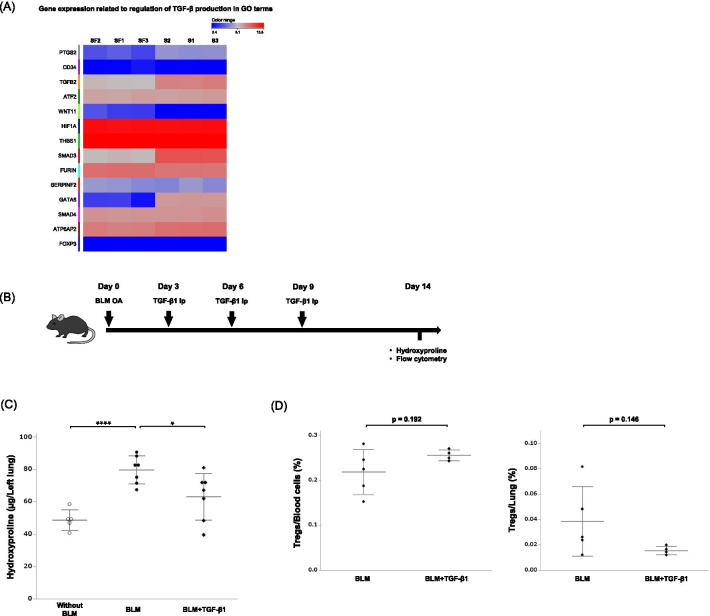
Effect of circulating TGF-β1 on Treg induction and BLM-induced pulmonary fibrosis. **A** The heatmap shows the gene expression related to the regulation of TGF-β production between S-MSCs (S1-3) and SF-MSCs (SF1-3). Each row represents a sample, and each line represents an expressed gene. Red color indicates upregulation and blue color indicates downregulation. **B** Experimental scheme of systemic TGF-β1 supplementation. ip, intraperitoneal injection. **C** The hydroxyproline content in the murine left lung (*n* = 5–7 per group). **D** The percentages of blood or lung Tregs in BLM-treated mice with or without TGF-β1 injection (*n* = 4–5 per group). Data are presented as means ± SD. *P < 0.05. ****P < 0.001

MSCs have been reported to produce TGF-β in vitro [5]. In our study, although we observed increased serum TGF-β1 levels in the SF-MSC group (Fig. 3F), RNA sequence analysis did not indicate a higher expression of TGF-β1 related genes in SF-MSCs compared with that in S-MSCs (Fig. 5A). This result was also replicated in the experiment using ELISA (Additional file 8). Next, to investigate the TGF-β1 production ability of MSCs in fibrotic lung environment, we stimulated MSCs with damage-associated molecular pattern (DAMP) molecules in vitro. To provide DAMP stimulus, we used a co-culture system between MSCs and bleomycin-induced fibrotic murine lung homogenates. Interestingly, DAMP stimulus decreased intracellular TGF-β1 production in MSCs, with changes milder in SF-MSCs (− 20.7% ± 5.9%) compared with S-MSCs (− 27.4% ± 4.6%). Furthermore, to evaluate the effect of upregulated circulating TGF-β1 in BLM-induced pulmonary fibrosis, mice were systemically supplemented with recombinant TGF-β1 (Fig. 5B). Supplementation with circulating TGF-β1 attenuated pulmonary fibrosis without increasing the number of blood and lung Tregs (Fig. 5C, D).

### Requirement of Treg induction for the antifibrotic effect of SF-MSCs

**Fig. 6:**
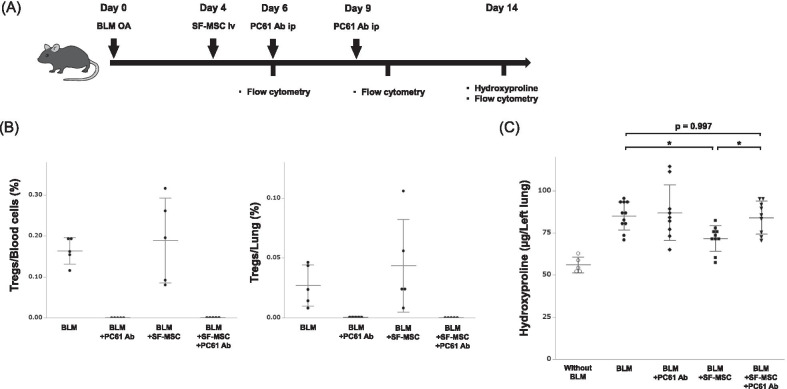
Tregs depletion abolished the antifibrotic effect of SF-MSCs during BLM-induced pulmonary fibrosis. **A** Experimental scheme of Treg depletion using the PC61 Ab in vivo. iv, intravenous injection. ip, intraperitoneal injection. **B** The percentage of blood or lung Tregs at 14 days after BLM OA in mice treated with or without PC61 Ab and SF-MSCs (*n* = 5 per group). **C** The hydroxyproline content in each group (*n* = 5–11 per group). Data are presented as means ± SD. *P < 0.05

To investigate whether increased Treg numbers play a role in the antifibrotic effect of SF-MSC treatment, we administered PC61 antibody, which functionally depletes Tregs, or an isotype control antibody to the BLM-treated mice (Fig. 6A). PC61 antibody administration successfully depleted Tregs in as early as 1 h, regardless of SF-MSC treatment (Additional file 10 and Fig. 6B). Complete depletion of Tregs using the PC61 antibody resulted in loss of the potential antifibrotic ability of SF-MSCs in BLM-induced pulmonary fibrosis (Fig. 6C).

## Discussion

**Fig. 7:**
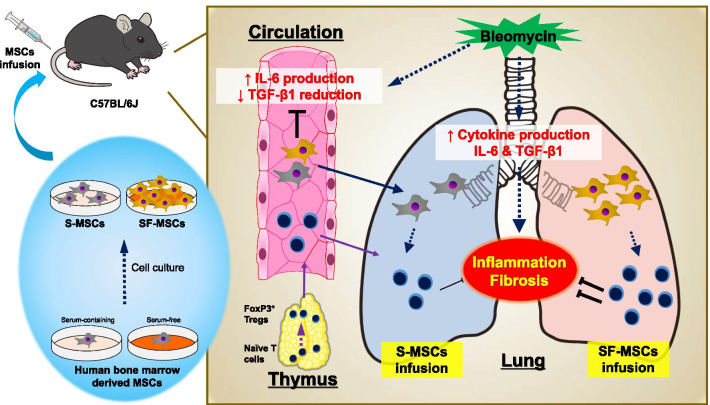
Summary of the study. Serum-free conditions promote cell proliferation potential of MSCs in vitro and intrapulmonary engraftment of intravenous delivered MSCs in vivo. SF-MSCs correct cytokine disruptions (e.g., IL-6 production in serum or BALF) caused by BLM administration. Furthermore, SF-MSCs lead to efficiently inhibition of BLM-induced lung inflammation and fibrosis by promoting induction of Tregs into the lung compared with S-MSCs

In this study, we showed that serum-free media increased proliferative capacity of MSCs and transformed the gene expression profiling of MSCs in vitro. *S*erum-free media also increased lung engraftment of intravenously administered MSCs in BLM-induced pulmonary fibrosis model mice. In addition, intravenously administered MSCs abolished the reduction in serum TGF-β1 and the increase in IL-6 in both the serum and the BALF caused by BLM treatment, which causally ameliorated BLM-induced pulmonary inflammation and fibrosis more effectively than the administration of S-MSCs. Finally, we found that treatment of SF-MSCs increased the number of murine thymus, blood, and lung Tregs, and depletion of the increased Tregs leads to abolish antifibrotic effect of SF-MSCs in vivo. Thus, this study showed that SF-MSCs suppress murine bleomycin-induced pulmonary fibrosis by enhancing regulatory T cell induction (Fig. 7).

We performed in vivo experiments using non-autologous MSCs. MSCs express low levels of major histocompatibility complex (MHC) class I and class II and do not express CD40, CD80, and CD86, which are necessary for T cell activation. Therefore, non-autologous MSCs do not induce lymphocyte proliferation when co-cultured with donor-derived lymphocytes that do not match their HLA type and can be transplanted across the MHC barrier safely [26, 27]. In fact, in recent years, more human clinical trials used non-autologous MSCs rather than autologous MSCs due to less difficulty of obtaining cells [28]. In addition, non-autologous MSCs have been administered to patients with graft-versus-host disease (GVHD) in clinical practice after safety confirmations of non-autologous MSCs transplantation in clinical trials [29]. Moreover, the use of autologous MSCs has some limitations. First, the use of autologous MSCs isolated from the patient may also adversely affect cell quality, depending on the patient’s age or disease status [30, 31]. In lung disease, it has been reported that transplantation of bone marrow-derived autologous MSCs resulted in reduced therapeutic efficiency compared with non-autologous transplantation in acute respiratory distress syndrome (ARDS) model mice [32]. This problem can be avoided by using allogeneic MSCs isolated from young healthy donors. Second, in vitro expansion of autologous MSCs can take several weeks to obtain enough cells for administration, making this therapeutic approach difficult for the early treatment in acute disease onset. Allogeneic MSCs, once obtained, cryopreserved and stocked, can be immediately administered to the patients when needed. For these reasons, non-autologous MSCs are a promising alternative to autologous MSCs with multiple advantages. Thus, our experimental procedure using non-autologous MSCs is a reasonable approach for clinical use.

We observed that SF-MSC administration increased the number of Tregs in both the blood and lungs more strongly than S-MSC administration. This increase in the number of Tregs preceded the antifibrotic effect of SF-MSCs, which was diminished by Treg depletion using Treg-specific antibodies. Increases in Tregs after systemic administration of S-MSCs have been previously reported in animal studies and in human clinical trials [33, 34]. Tregs show immunomodulatory functions that suppress inflammation and repair injured tissues, resulting in resolution of fibrosis and recovery of organ function [35]. Although the role and function of Tregs in the fibrotic cascades in patients with IPF remain unclear, several reports suggest Tregs play protective roles in BLM-treated mice, which is the most reliable animal model for preclinical experiments on IPF [36–38]. In an animal model of lipopolysaccharide-induced acute lung injury, Tregs are reported to be necessary for recovery from inflammation and for lung tissue repair to promote lung epithelial cell proliferation [39]. Further, Tregs can protect against pulmonary fibrosis following bacterial infection [40]. Based on these findings, we concluded that an increase in Tregs is central to the anti-inflammatory and antifibrotic effects of SF-MSC administration.

BLM administration to the airway resulted in reduced serum TGF-β1 and increased serum and BALF IL-6 levels. We found both changes were strongly inhibited by SF-MSC administration. Tregs, specifically cells expressing the transcription factor Foxp3, are induced to differentiate from naïve CD4 + T cells by TGF-β; this induction of differentiation was completely inhibited in the presence of the pro-inflammatory cytokine IL-6 [41]. We thus tested whether systemic supplementation of reduced TGF-β1 in BLM-treated mice mimicked the favorable effects of SF-MSC transfer. We found that systemic supplementation with TGF-β1 ameliorated pulmonary fibrosis without increasing the number of Tregs, suggesting that SF-MSC-mediated Treg induction did not solely depend on the upregulation of systemic TGF-β1, but also depended on other factors such as strong IL-6 suppression. Although TGF-β1 in the lung plays key roles in promoting pulmonary fibrosis [42, 43], circulating TGF-β1 has pleiotropic effects, including anti-inflammatory and immunosuppressive effects [44]. Thus, the inhibited reduction in serum TGF-β1 observed in SF-MSC-treated mice might serve as one mechanism underlying the anti-inflammatory and antifibrotic effects of SF-MSC administration. Regarding IL-6, the highest expressed cytokine in BALF obtained from both human and murine fibrotic lungs [45], SF-MSC administration significantly inhibited IL-6 levels in both the serum and BALF compared with S-MSC administration. Notably, the suppressive effect of SF-MSCs on circulating IL-6 at day 14, regarded as the late phase of BLM-induced pulmonary fibrosis, was extremely strong, resulting in levels as low as those in control mice without BLM treatment. Interestingly, IL-6 plays a biphasic role in the pathogenesis of pulmonary fibrosis. For instance, IL-6 blockade using an IL-6-neutralizing antibody in the early or late phase of BLM-induced pulmonary fibrosis resulted in the exacerbation or amelioration of pulmonary fibrosis, respectively [46]. Seen from this viewpoint, SF-MSC treatment in the current study suppressed the serum and BALF levels of IL-6, especially in the late phase, suggesting that SF-MSC administration reasonably ameliorates established pulmonary fibrosis.

Our data demonstrate MSCs cultured in serum-free conditions showed improved lung engraftment compared with those cultured in serum-containing media. Further, RNA sequence analysis and cytokine measurement of the medium supernatant demonstrated no difference in TGF-β1 production between S-MSCs and SF-MSCs. Thus, the differences in the regulation of cytokines, Treg induction, and antifibrotic effect between SF-MSCs and S-MSCs may be due to the presence of a larger number of engrafted SF-MSCs in the lung compared with that of S-MSCs. This inference is also supported by results where SF-MSCs administered intraperitoneally or subcutaneously neither settle in the murine lung nor inhibit lung fibrosis. Conversely, intravenous administration results in the overwhelming majority of MSCs in the murine lungs [47, 48], which was replicated in our study. Moreover, MSCs protect against lung injury and fibrosis both in vitro and in vivo through a paracrine anti-inflammatory mechanism [7, 49]. In addition, in a renal ischemia–reperfusion injury rat model, it has been reported that intra-arterial administration of MSCs, via the renal artery, enhanced engraftment of MSCs in kidneys and the therapeutic effect compared with intravenous administration [50]. These findings suggest that the protective effect of MSCs on pulmonary fibrosis may require intrapulmonary engraftment of delivered MSCs, and that culturing them in serum-free medium enhances this effect. This enhancement might be partly due to the downregulation of positive apoptosis process such as *Casp1* (Caspase 1; *P* = 0.025), *FADD* (Fas-associated via death domain; *P* = 0.017) and *MAP3K5* (Mitogen-activated protein kinase kinase kinase 5; *P* = 0.001) in SF-MSCs compared with S-MSCs, as shown by RNA sequencing analysis.

To apply SF-MSCs to human diseases, culturing in serum-free media resulted in significantly higher proliferation of human MSCs compared with culturing in the classical serum-containing media, while maintaining the MSC properties. Thus, serum-free media allow obtaining the required dose of therapeutic cells for transfer into the recipient in the short term. In addition, using SF-MSCs is free from the transmission of unknown pathogens and immune responses typically caused by S-MSCs. Another concern with intravenous MSC administration is pulmonary embolism, especially in lungs with inflammation and fibrosis [51]. In a previous preclinical report, intravenous administration of higher dose bone marrow-derived-MSCs caused aggregation in the microcirculation and pulmonary embolism, resulting in respiratory and circulatory failure in mice [52]. Because of their enhanced effects, SF-MSCs treatment requires a smaller number of cells, leading to risk avoidance of this embolism event. In previous clinical trials using MSCs for human pulmonary fibrosis, the dose of administered MSCs was approximately 2.9 × 10^5^–2.9 × 10^6^/infusion per kg (calculated at 70 kg per body) [9, 10]. In addition, human allogenic MSCs (Temcell HS Injection, JCR Pharmaceuticals, Ashiya, Japan) have been approved to treat GVHD in clinical practice at a dose of 2.0 × 10^6^ MSCs/infusion per kg twice per week [29]. In this study, we administered approximately 4.7 × 10^6^ SF-MSCs/infusion per kg to mice, which corresponds to about two-thirds to one-fortieth dose reported in preclinical studies of MSCs in BLM-induced pulmonary fibrosis model mice [53]. Thus, compared with previous preclinical studies, the dose of MSCs in our study protocol was more in line with those in previous clinical reports, indicating this study can be applied in clinical practice. Given that human clinical trials with S-MSC treatment have shown a protective effect against the fibrotic process in patients with IPF [10], SF-MSCs could be a more useful therapeutic tool in future IPF therapy.

Besides patients with IPF, SF-MSCs may possess therapeutic potential for patients with coronavirus disease 2019 (COVID-19) complicated by ARDS. Animal and human studies have confirmed that MSC-based therapy improves the respiratory status of recipients with ARDS [54]. COVID-19 ARDS is triggered by a cytokine storm in which IL-6 plays a key role; thus, an anti-IL-6 receptor antibody showed favorable effects in patients with COVID-19 ARDS [55]. Furthermore, in addition to MSC-based therapy, Treg-based therapy has been reported as effective in multiple preclinical models of ARDS [56]. The number of Tregs in peripheral blood, which migrate to the lungs to prevent lung tissue damage [57], was significantly decreased in patients with severe COVID-19 compared with that in healthy controls [58, 59]. Based on these findings, Treg-based therapy is expected to be an effective treatment for COVID-19 ARDS. However, the Tregs isolated from each patient require 2–3 weeks for ex vivo expansion to achieve sufficient quantities for clinical use [60]. Alternatively, MSCs can be isolated from multiple tissues [4] and quickly expanded to clinically relevant numbers under serum-free culture conditions. Moreover, treatment with S-MSCs in subjects with COVID-19 ARDS showed a positive response without serious adverse events [61]. With no end in sight to the ongoing COVID-19 pandemic, a clinical trial to evaluate the potential therapeutic role of SF-MSCs in COVID-19 ARDS would be worth conducting.

## Conclusions

SF-MSCs significantly suppressed BLM-induced pulmonary inflammation and fibrosis through enhanced induction of Tregs into the lungs and corrected the dysregulated cytokine balance. Besides their remarkable therapeutic effects, MSCs cultured in serum-free media pose reduced risks compared with cells cultured in serum-containing media in clinical settings. Administration of ex vivo expanded SF-MSCs could, thus, be an effective therapeutic strategy to treat pulmonary fibrosis.

## Supplementary Information

**Additional file 1:** Representative flow cytometry analysis related to the defined positive and negative MSC surface markers on MSCs cultured in DMEM with 10% FBS (S-MSC) or in serum-free STK2 medium (SF-MSC).

**Additional file 2:** Fluorescence microscopic ex vivo images of engrafted DiI-labeled SF-MSCs (yellow arrows) in murine lungs on the day after injection.

**Additional file 3:** Cytokine levels in BALF before BLM OA or at 7 or 14 days after BLM OA (n = 5–8 per group). Data are presented as the mean ± SD for normal distribution (IL-13), or as the median with interquartile range for non-normal distribution (TNF-α, IL-4, IL-5, IL-10).

**Additional file 4:** Cytokine levels in serum before BLM OA or at 4, 7, 14, or 21 days after BLM OA (n = 3–7 per group). Data are presented as the median with interquartile range.

**Additional file 5:** Changes in body weight in mice after BLM OA with treatment of MSCs. BLM OA was performed at day 0, and MSCs were injected via the tail vein at a dose of 1.0 × 10^5^ cells/mouse in 100 µL of PBS 4 days after BLM OA. Mice in the PBS with BLM group were injected with 100 µL of PBS intravenously instead of MSCs. Mice in the without-BLM group were aspirated with PBS alone instead of BLM and were injected with PBS intravenously. On the indicated days, data are expressed as a percentage of the mean weight in each group measured on the first day of the experiment. Data are presented as means ± SD for 4–5 mice per group.

**Additional file 6:** Hydroxyproline levels in the murine left lung at 14 days after BLM OA. At 4 days after BLM OA, mice were further subjected to subcutaneous (sc) or intraperitoneal (ip) administration of SF-MSCs at a dose of 1 × 10^5^ in 100 µL of PBS. Mice without MSC administration were used as controls. Data are presented as the means ± SD (n = 5–8 per group). NS, not significant.

**Additional file 7:** Representative flow cytometry analysis on engrafted DiI-labeled MSCs in the murine lung. DiI-labeled SF-MSCs were injected into mice subcutaneously (sc) or intraperitoneally (ip) at a dose of 2.0 × 10^5^ cells/mouse in 100 µL of PBS at 4 days after BLM OA. On the day after DiI-labeled SF-MSC injection, MSCs engrafted in murine lungs were measured using flow cytometry.

**Additional file 8:** S-MSCs or SF-MSCs were seeded into 24-well plates at a density of 5 × 10^3^ cells/well. These MSCs were cultured in serum-free DMEM for 48 h. The supernatants of the culture medium were collected at 6, 12, 24, or 48 h, and the TGF-β1 concentration in the supernatants was measured using an ELISA kit. Data are expressed as concentration of TGF-β1 per each live 100,000 MSCs in the cell culture media. Data are presented as means ± SD (n = 4 per group). On the indicated hours, there was no statistically significant difference in TGF-β1 between the two MSC groups.

**Additional file 9:** Lung homogenate was generated from the left lung of mice at 7 days after PBS OA (DAMP- group) or BLM OA (DAMP+ group). Upper inserts (pore size, 0.4 μm; Corning) with cultured lung homogenates were dipped into the basal plate of MSCs (1.0 × 10^5^ cells/well) cultured in DMEM with 10% FBS (S-MSCs) or in serum-free STK2 media (SF-MSCs). After 72 hours, the MSCs were harvested, and intracellular proteins were extracted for TGF-β1 measurement. TGF-β1 was measured using an ELISA kit. Data were calculated as a TGF-β1 per each number of live MSCs in the culture media, and expressed as a percentage of the mean in DAMP- group. Data are presented as means ± SD (n = 3 per group).

**Additional file 10:** Representative flow cytometry analysis of the Tregs fraction (red box) in murine blood or lung CD4+ T cells at 6, 10, or 14 days after BLM OA. BLM-administered mice were treated with or without SF-MSC plus Treg depletion Ab (PC61) as shown in Figure 6A.

## Data Availability

The data that support the findings of this study are available from the corresponding author upon reasonable request.

## Abbreviations

ARDS: Acute respiratory distress syndrome
BALF: Bronchoalveolar lavage fluid
BLM: Bleomycin
COVID-19: Coronavirus disease 2019
DAMP: Damage-associated molecular pattern
DEG: Differentially expressed gene
DMEM: Dulbecco’s modified Eagle medium
FBS: Fetal bovine serum
FVC: Forced vital capacity
GM-CSF: Granulocyte macrophage-colony stimulating factor
GVHD: Graft-versus-host disease
HIF: Hypoxia-inducible factor
IFN: Interferon
IL: Interleukin
IPF: Idiopathic pulmonary fibrosis
ISCT: International Society for Cellular Therapy
MSC: Mesenchymal stromal cell
OA: Oropharyngeal aspiration
PBS: Phosphate-buffered saline
RAS: Renin angiotensin system
S-MSCs: MSCs cultured in serum-containing medium
SF-MSCs: MSCs cultured in serum-free medium
TGF: Transforming growth factor
TNF: Tumor necrosis factor
Treg: Regulatory T cell
