# Supplementary figures and images for: Human bone marrow-derived mesenchymal stromal cells cultured in serum-free media demonstrate enhanced antifibrotic abilities via prolonged survival and robust regulatory T cell induction in murine bleomycin-induced pulmonary fibrosis
Source: Stem Cell Res Ther. 2021 Sep 16;12:506. doi: 10.1186/s13287-021-02574-5 (PMC8444523; doi:10.1186/s13287-021-02574-5)

## Slide 1
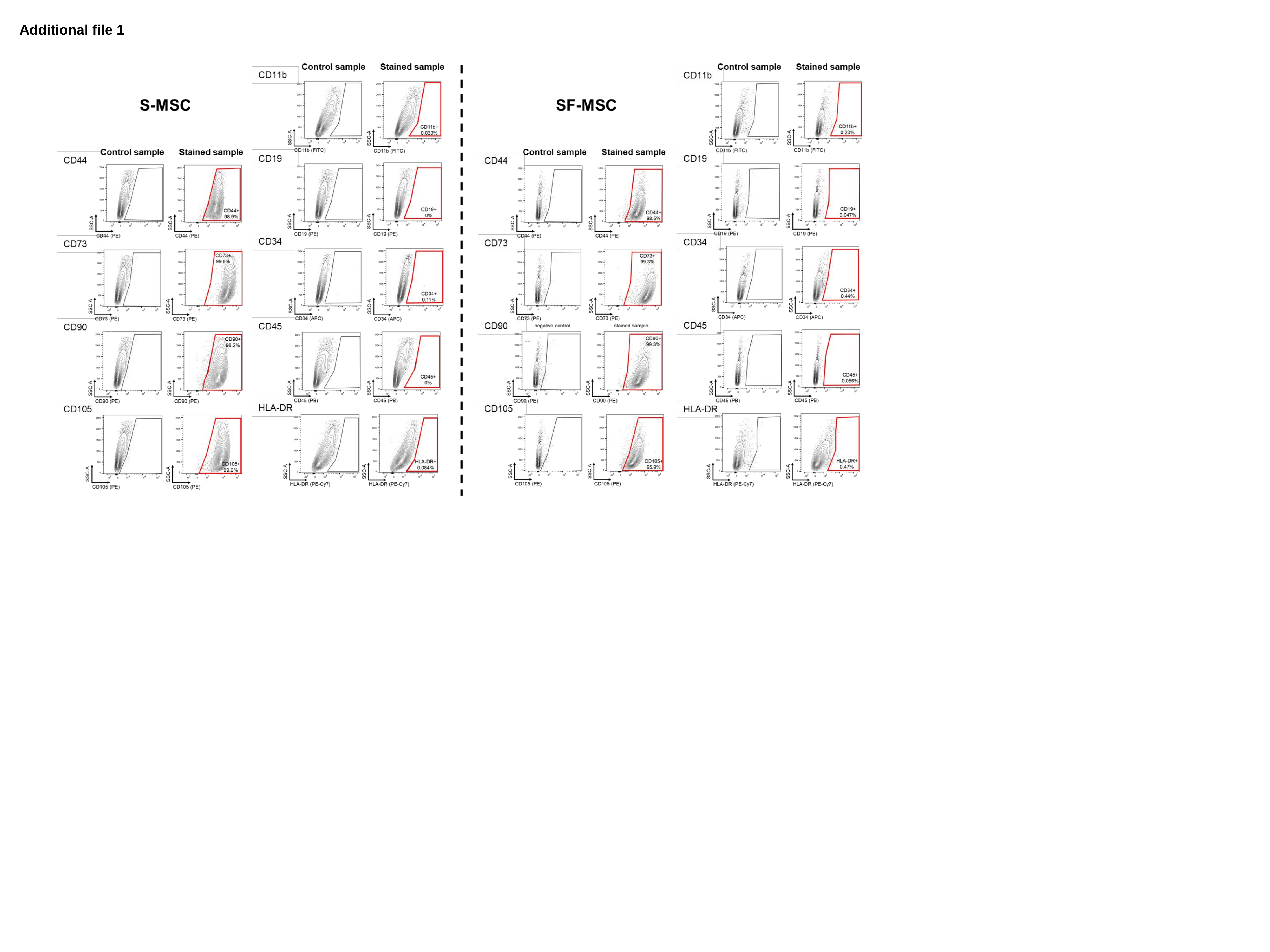

Additional file 1

Supplement: Supplementary file 1 — Additional file 1: Representative flow cytometry analysis related to the defined positive and negative MSC surface markers on MSCs cultured in DMEM with 10% FBS (S-MSC) or in serum-free STK2 medium (SF-MSC). [file 13287_2021_2574_MOESM1_ESM.pptx]

## Slide 1
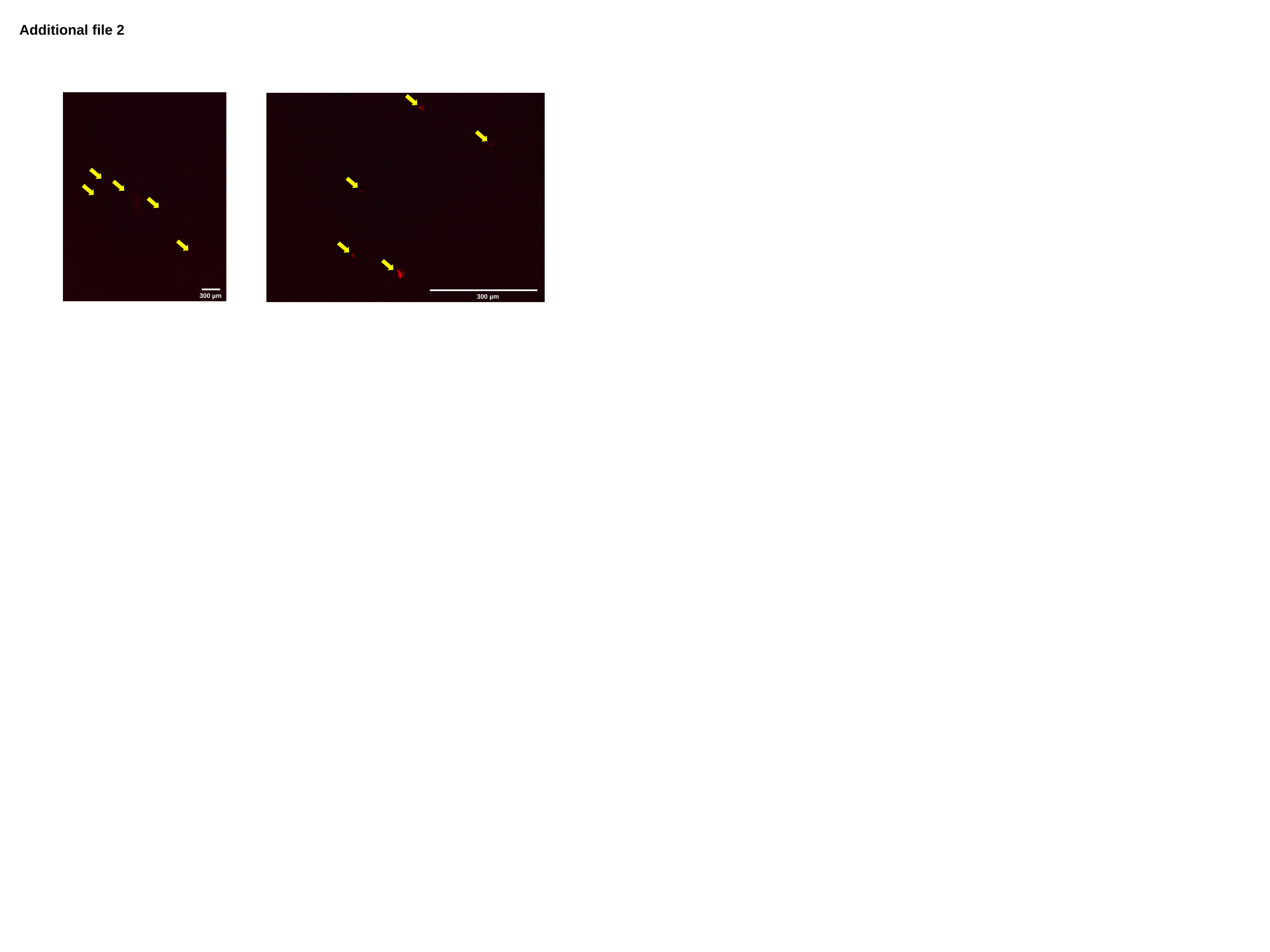

Additional file 2
300 µm
300 µm

Supplement: Supplementary file 2 — Additional file 2: Fluorescence microscopic ex vivo images of engrafted DiI-labeled SF-MSCs (yellow arrows) in murine lungs on the day after injection. [file 13287_2021_2574_MOESM2_ESM.pptx]

## Slide 1
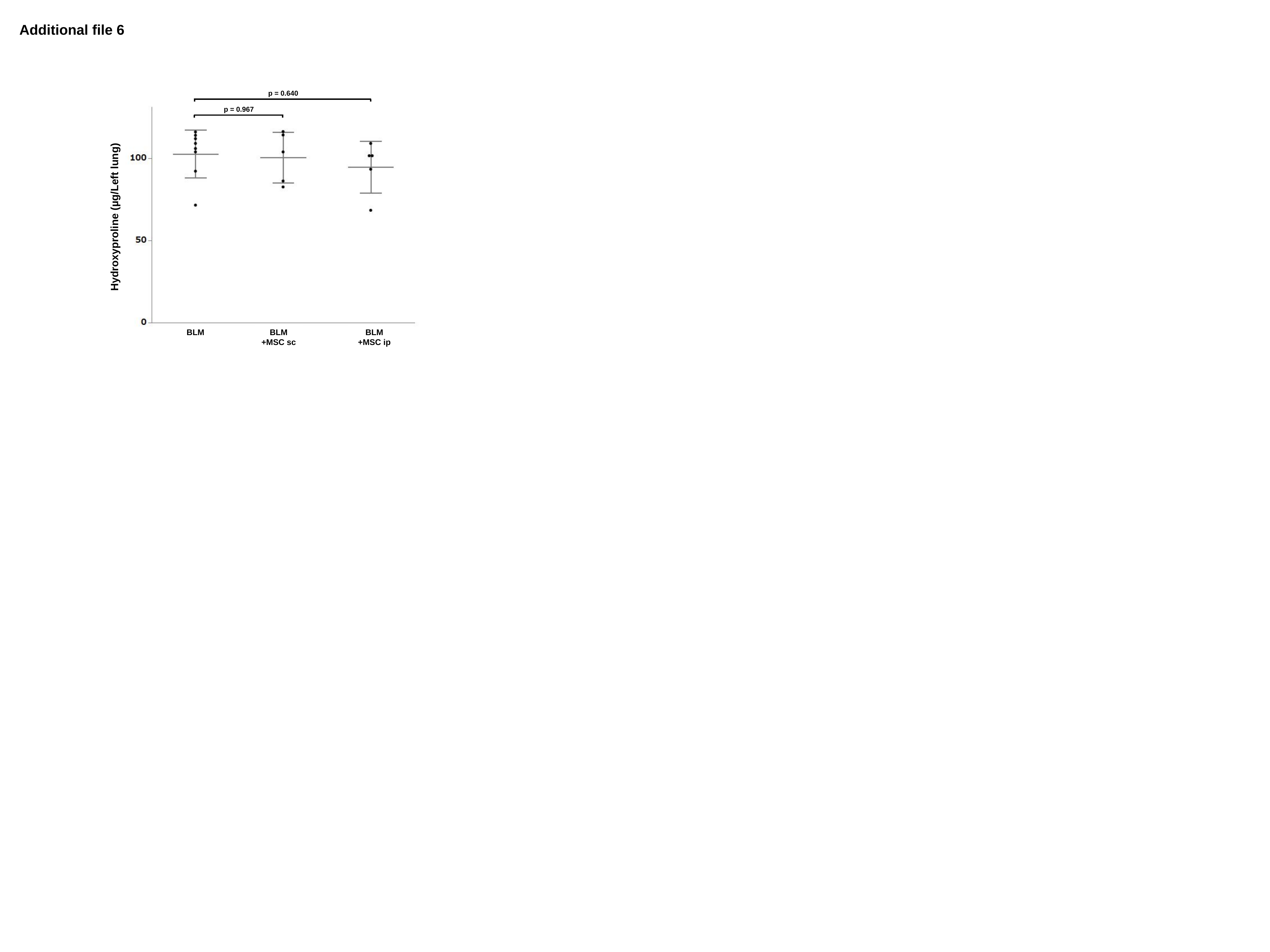

Additional file 6
p = 0.640
p = 0.967
Hydroxyproline (µg/Left lung)
BLM
BLM
+MSC sc
BLM
+MSC ip

Supplement: Supplementary file 6 — Additional file 6: Hydroxyproline levels in the murine left lung at 14 days after BLM OA. At 4 days after BLM OA, mice were further subjected to subcutaneous (sc) or intraperitoneal (ip) administration of SF-MSCs at a dose of 1 × 10^5^ in 100 µL of PBS. Mice without MSC administration were used as controls. Data are presented as the means ± SD (n = 5–8 per group). NS, not significant. [file 13287_2021_2574_MOESM6_ESM.pptx]

## Slide 1
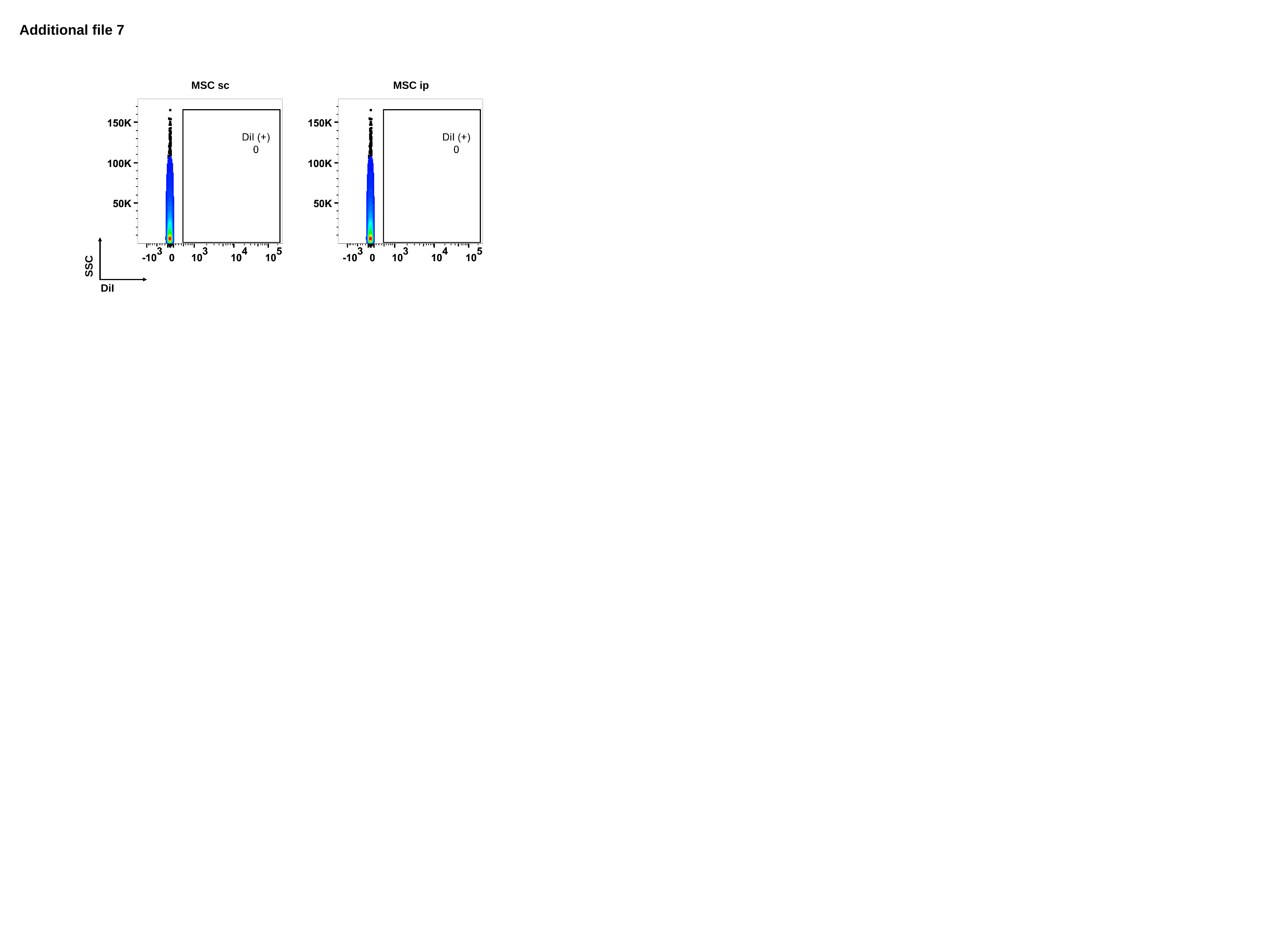

Additional file 7
MSC sc
MSC ip
SSC
DiI

Supplement: Supplementary file 7 — Additional file 7: Representative flow cytometry analysis on engrafted DiI-labeled MSCs in the murine lung. DiI-labeled SF-MSCs were injected into mice subcutaneously (sc) or intraperitoneally (ip) at a dose of 2.0 × 10^5^ cells/mouse in 100 µL of PBS at 4 days after BLM OA. On the day after DiI-labeled SF-MSC injection, MSCs engrafted in murine lungs were measured using flow cytometry. [file 13287_2021_2574_MOESM7_ESM.pptx]

## Slide 1
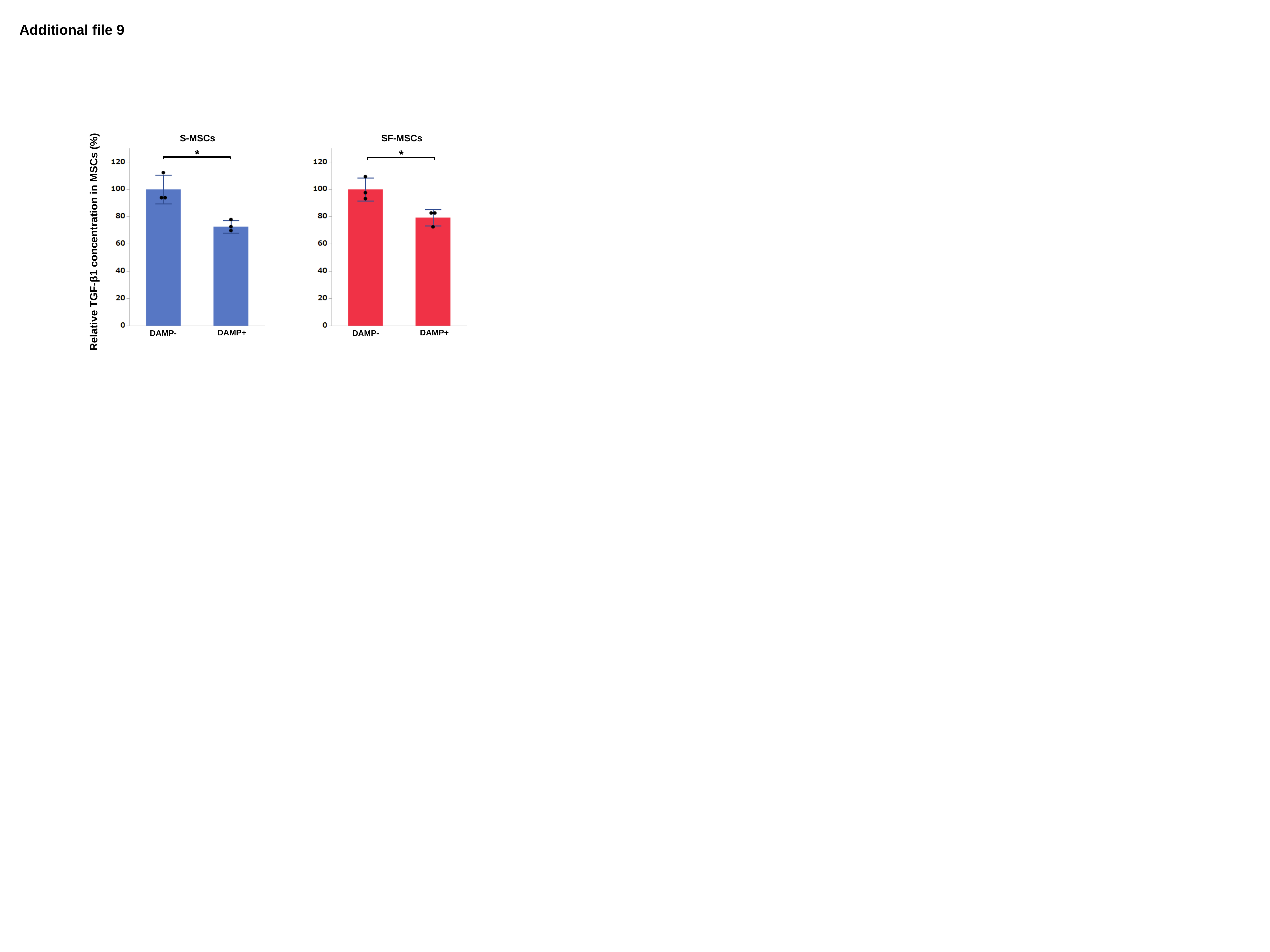

Additional file 9
S-MSCs
DAMP+
DAMP-
SF-MSCs
DAMP+
DAMP-
*
*
Relative TGF-β1 concentration in MSCs (%)

Supplement: Supplementary file 9 — Additional file 9: Lung homogenate was generated from the left lung of mice at 7 days after PBS OA (DAMP- group) or BLM OA (DAMP+ group). Upper inserts (pore size, 0.4 μm; Corning) with cultured lung homogenates were dipped into the basal plate of MSCs (1.0 × 10^5^ cells/well) cultured in DMEM with 10% FBS (S-MSCs) or in serum-free STK2 media (SF-MSCs). After 72 hours, the MSCs were harvested, and intracellular proteins were extracted for TGF-β1 measurement. TGF-β1 was measured using an ELISA kit. Data were calculated as a TGF-β1 per each number of live MSCs in the culture media, and expressed as a percentage of the mean in DAMP- group. Data are presented as means ± SD (n = 3 per group). [file 13287_2021_2574_MOESM9_ESM.pptx]

## Slide 1
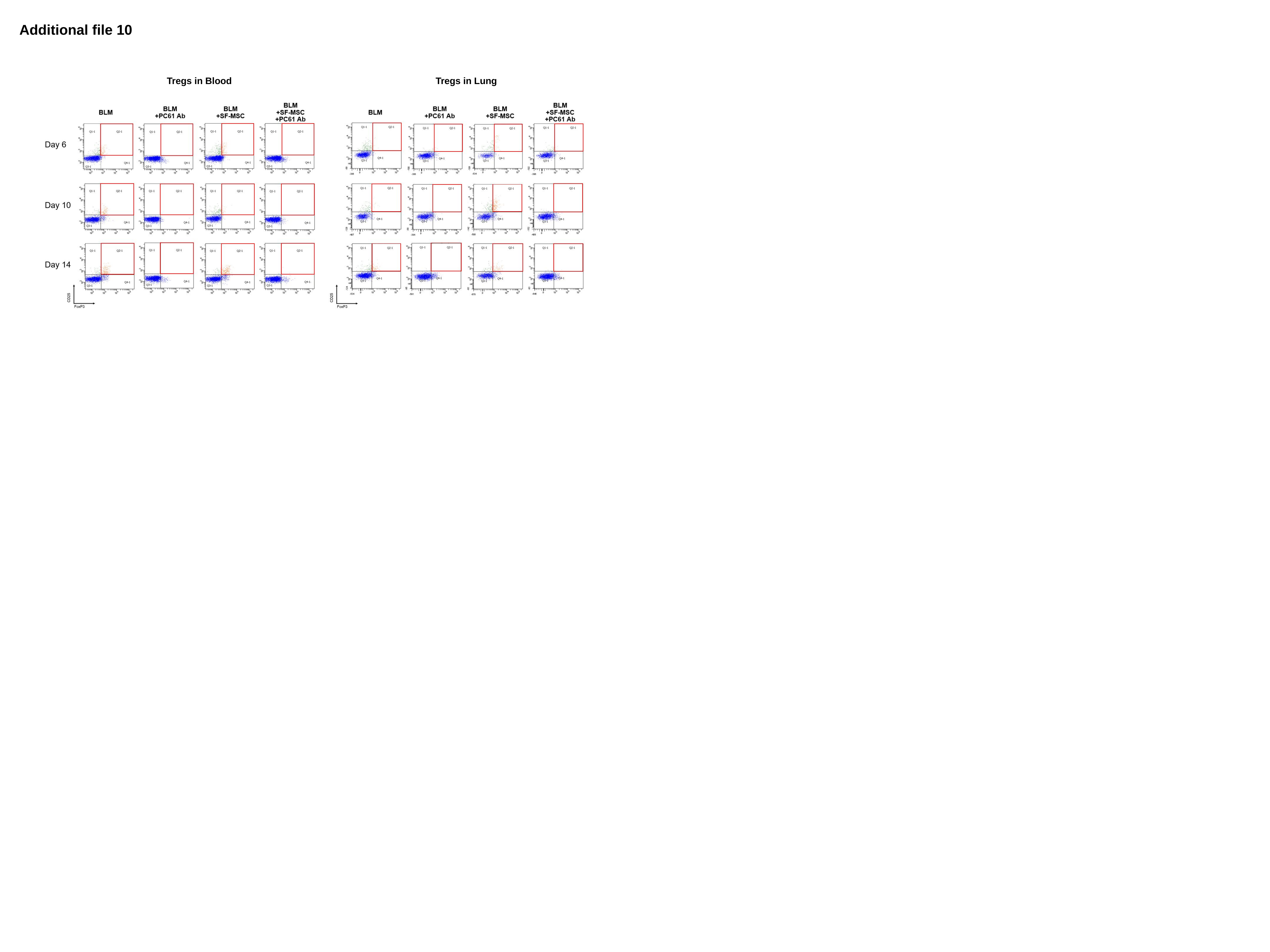

Additional file 10
Tregs in Lung
Tregs in Blood

Supplement: Supplementary file 10 — Additional file 10: Representative flow cytometry analysis of the Tregs fraction (red box) in murine blood or lung CD4+ T cells at 6, 10, or 14 days after BLM OA. BLM-administered mice were treated with or without SF-MSC plus Treg depletion Ab (PC61) as shown in Figure 6A. [file 13287_2021_2574_MOESM10_ESM.pptx]
