# Supplementary material for: Human bone marrow-derived mesenchymal stromal cells cultured in serum-free media demonstrate enhanced antifibrotic abilities via prolonged survival and robust regulatory T cell induction in murine bleomycin-induced pulmonary fibrosis
Source: Stem Cell Res Ther. 2021 Sep 16;12:506. doi: 10.1186/s13287-021-02574-5 (PMC8444523; doi:10.1186/s13287-021-02574-5)
Supplement: Supplementary file 3 — Additional file 3: Cytokine levels in BALF before BLM OA or at 7 or 14 days after BLM OA (n = 5–8 per group). Data are presented as the mean ± SD for normal distribution (IL-13), or as the median with interquartile range for non-normal distribution (TNF-α, IL-4, IL-5, IL-10). [file 13287_2021_2574_MOESM3_ESM.pptx]

## Slide 1
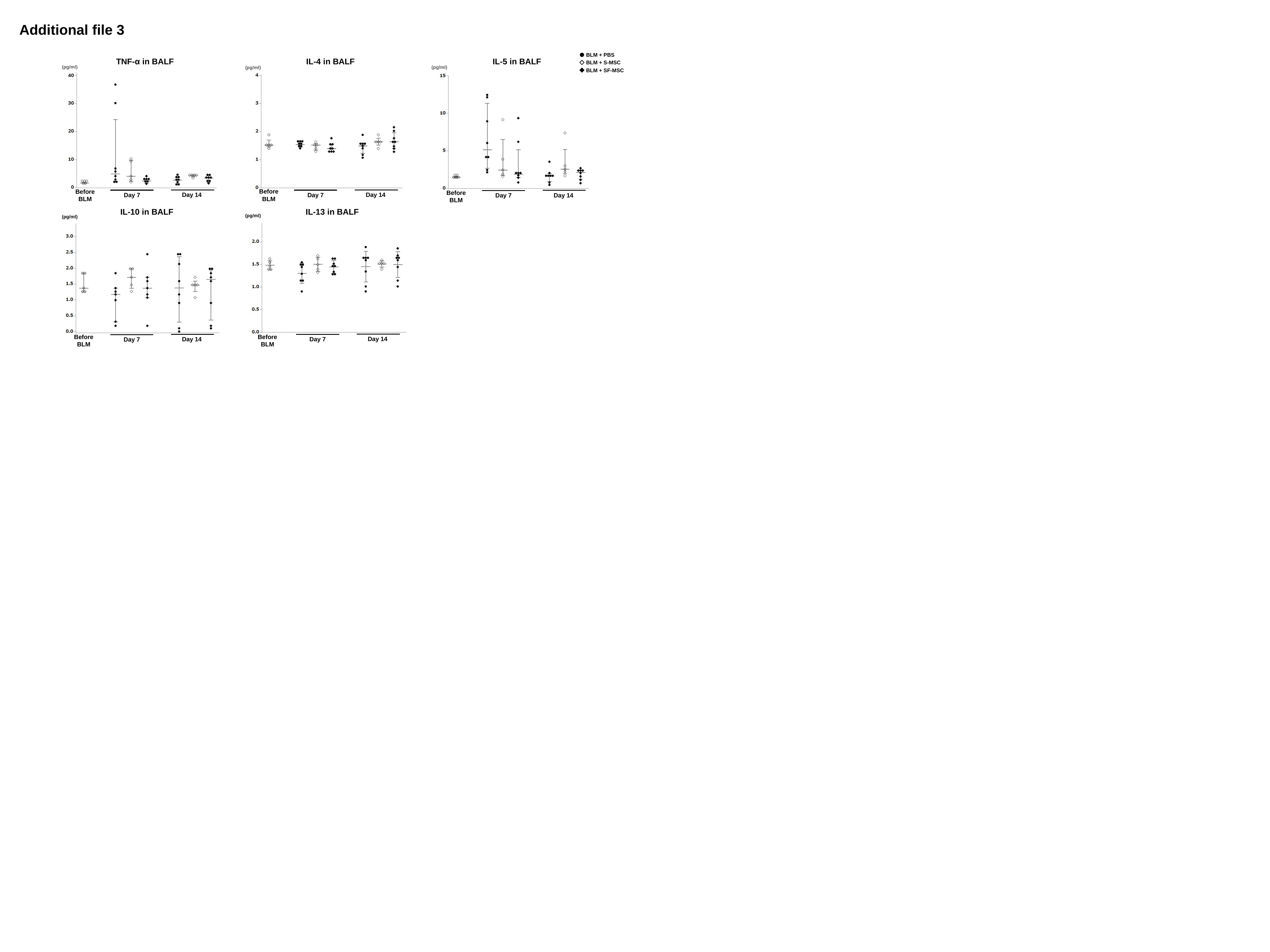

Additional file 3
BLM + PBS
BLM + S-MSC
BLM + SF-MSC
TNF-α in BALF
(pg/ml)
Before
BLM
Day 14
Day 7
IL-4 in BALF
(pg/ml)
Before
BLM
Day 14
Day 7
IL-5 in BALF
(pg/ml)
Before
BLM
Day 14
Day 7
IL-10 in BALF
(pg/ml)
Before
BLM
Day 14
Day 7
IL-13 in BALF
(pg/ml)
Before
BLM
Day 14
Day 7
