# Supplementary material for: Human bone marrow-derived mesenchymal stromal cells cultured in serum-free media demonstrate enhanced antifibrotic abilities via prolonged survival and robust regulatory T cell induction in murine bleomycin-induced pulmonary fibrosis
Source: Stem Cell Res Ther. 2021 Sep 16;12:506. doi: 10.1186/s13287-021-02574-5 (PMC8444523; doi:10.1186/s13287-021-02574-5)
Supplement: Supplementary file 4 — Additional file 4: Cytokine levels in serum before BLM OA or at 4, 7, 14, or 21 days after BLM OA (n = 3–7 per group). Data are presented as the median with interquartile range. [file 13287_2021_2574_MOESM4_ESM.pptx]

## Slide 1
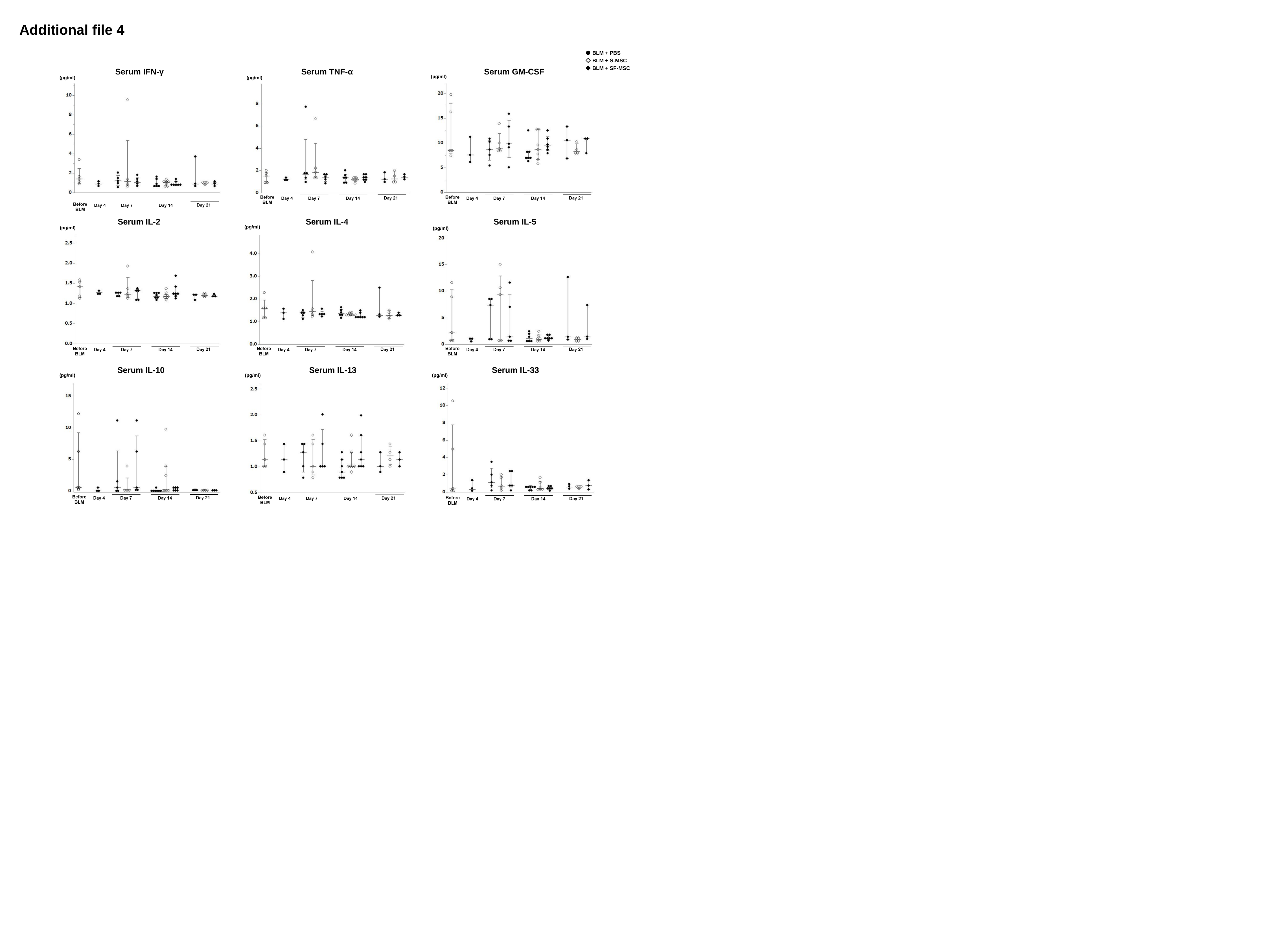

Additional file 4
BLM + PBS
BLM + S-MSC
BLM + SF-MSC
Serum IFN-γ
(pg/ml)
Serum TNF-α
(pg/ml)
Serum GM-CSF
(pg/ml)
Serum IL-2
(pg/ml)
Serum IL-4
(pg/ml)
Serum IL-5
(pg/ml)
Serum IL-10
(pg/ml)
Serum IL-13
(pg/ml)
Serum IL-33
(pg/ml)
