# Supplementary material for: Human bone marrow-derived mesenchymal stromal cells cultured in serum-free media demonstrate enhanced antifibrotic abilities via prolonged survival and robust regulatory T cell induction in murine bleomycin-induced pulmonary fibrosis
Source: Stem Cell Res Ther. 2021 Sep 16;12:506. doi: 10.1186/s13287-021-02574-5 (PMC8444523; doi:10.1186/s13287-021-02574-5)
Supplement: Supplementary file 5 — Additional file 5: Changes in body weight in mice after BLM OA with treatment of MSCs. BLM OA was performed at day 0, and MSCs were injected via the tail vein at a dose of 1.0 × 10^5^ cells/mouse in 100 µL of PBS 4 days after BLM OA. Mice in the PBS with BLM group were injected with 100 µL of PBS intravenously instead of MSCs. Mice in the without-BLM group were aspirated with PBS alone instead of BLM and were injected with PBS intravenously. On the indicated days, data are expressed as a percentage of the mean weight in each group measured on the first day of the experiment. Data are presented as means ± SD for 4–5 mice per group. [file 13287_2021_2574_MOESM5_ESM.pptx]

## Slide 1
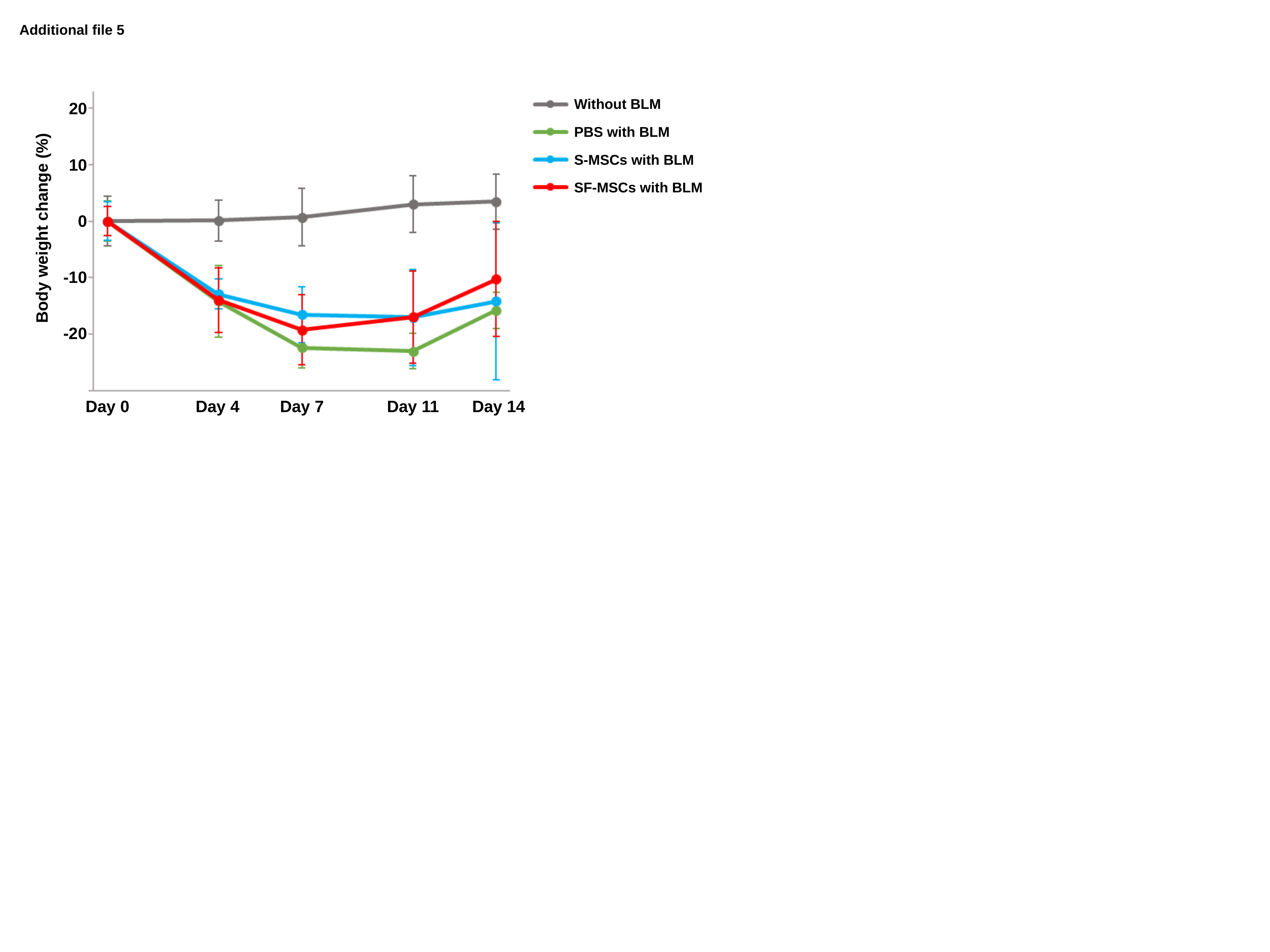

Additional file 5
Without BLM
20
PBS with BLM
S-MSCs with BLM
10
SF-MSCs with BLM
0
Body weight change (%)
-10
-20
Day 0
Day 4
Day 7
Day 11
Day 14
