# Supplementary material for: Human bone marrow-derived mesenchymal stromal cells cultured in serum-free media demonstrate enhanced antifibrotic abilities via prolonged survival and robust regulatory T cell induction in murine bleomycin-induced pulmonary fibrosis
Source: Stem Cell Res Ther. 2021 Sep 16;12:506. doi: 10.1186/s13287-021-02574-5 (PMC8444523; doi:10.1186/s13287-021-02574-5)
Supplement: Supplementary file 8 — Additional file 8: S-MSCs or SF-MSCs were seeded into 24-well plates at a density of 5 × 10^3^ cells/well. These MSCs were cultured in serum-free DMEM for 48 h. The supernatants of the culture medium were collected at 6, 12, 24, or 48 h, and the TGF-β1 concentration in the supernatants was measured using an ELISA kit. Data are expressed as concentration of TGF-β1 per each live 100,000 MSCs in the cell culture media. Data are presented as means ± SD (n = 4 per group). On the indicated hours, there was no statistically significant difference in TGF-β1 between the two MSC groups. [file 13287_2021_2574_MOESM8_ESM.pptx]

## Slide 1
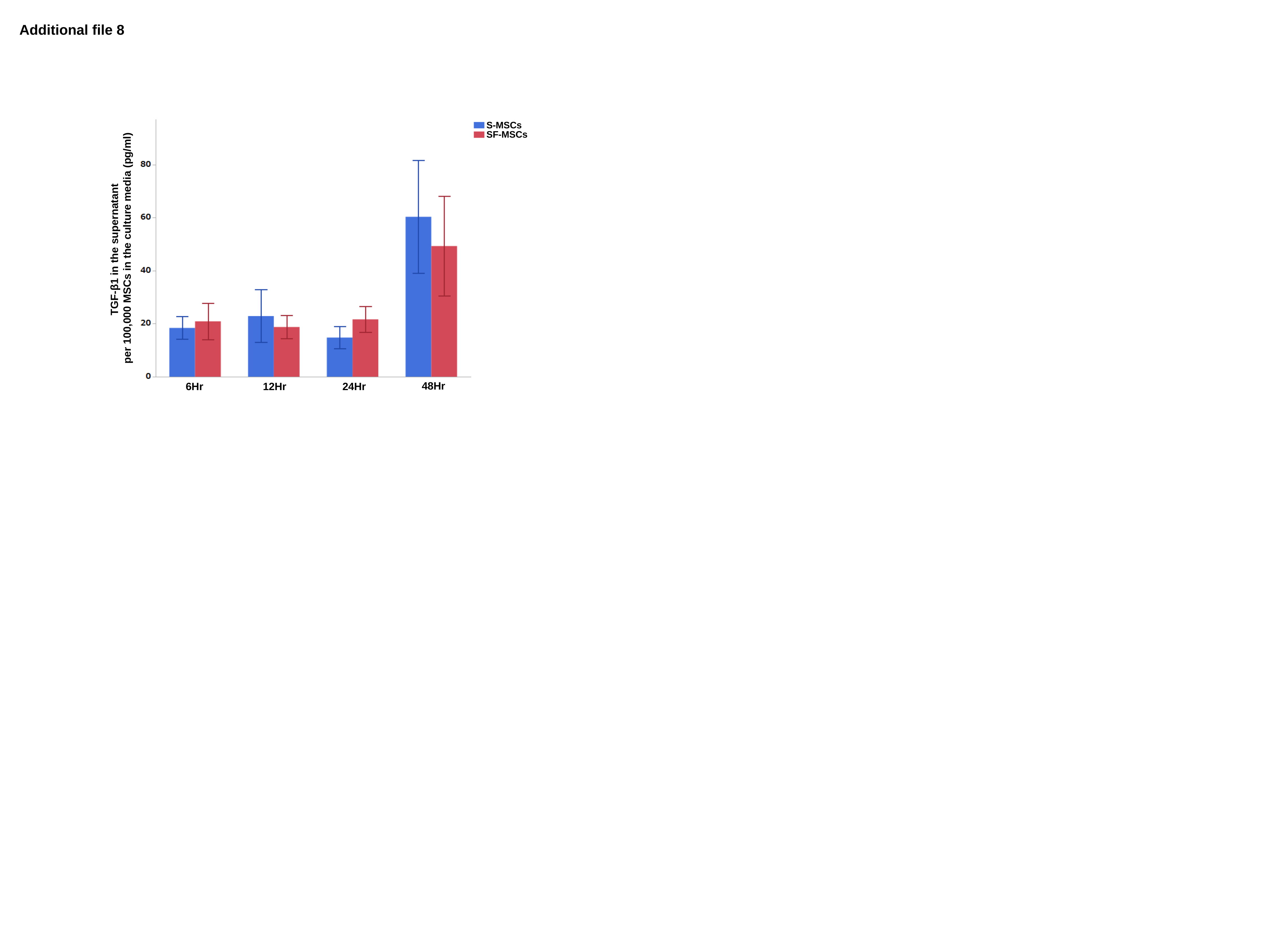

Additional file 8
S-MSCs
SF-MSCs
TGF-β1 in the supernatant
 per 100,000 MSCs in the culture media (pg/ml)
48Hr
6Hr
12Hr
24Hr
